# Chemistry in nanoconfined water†
†Electronic supplementary information (ESI) available: Details on the theory and methods, computational setup, and exhaustive description of the results. See DOI: 10.1039/c6sc04989c



**DOI:** 10.1039/c6sc04989c

**Published:** 2017-03-20

**Authors:** Daniel Muñoz-Santiburcio, Dominik Marx

**Affiliations:** a Lehrstuhl für Theoretische Chemie , Ruhr – Universität Bochum , 44780 Bochum , Germany . Email: daniel.munoz@theochem.rub.de

## Abstract

Nanoconfined liquids have extremely different properties from the bulk, which profoundly affects chemical reactions taking place in nanosolvation. Here, we present extensive *ab initio* simulations of a vast set of chemical reactions within a water lamella that is nanoconfined by mineral surfaces, which might be relevant to prebiotic peptide formation in aqueous environments. Our results disclose a rich interplay of distinct effects, from steric factors typical of reactions occurring in small spaces to a charge-stabilization effect in nanoconfined water at extreme conditions similar to that observed in bulk water when changing from extreme to ambient conditions. These effects are found to modify significantly not only the energetics but also the mechanisms of reactions happening in nanoconfined water in comparison to the corresponding bulk regime.

## Abstract 2

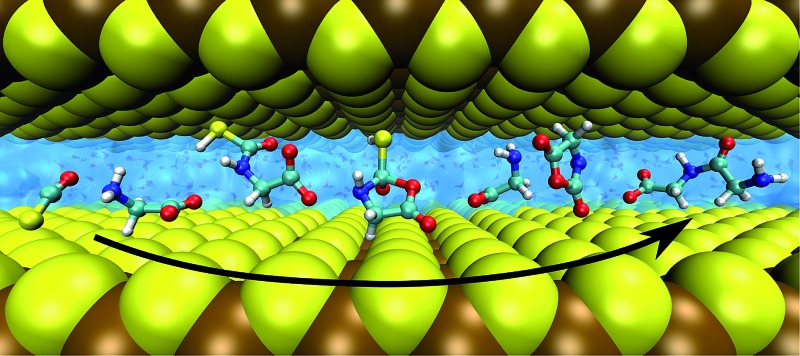
Nanoconfined liquids have extremely different properties from the bulk, which profoundly affects chemical reactions taking place in nanosolvation.

## 1 Introduction

Chemical reactions and physico-chemical processes in ultra-small spaces are receiving increasing attention due to their importance in biology,^1,2^ chemical synthesis^3,4^ and chemical analysis.^5^ While chemistry in microfluidic systems is now a very mature field,^6^ chemistry in the nanofluidic regime is yet an emerging field. Only in recent years have we seen stunning advances such as fabrication of reaction chambers at the nanoscale^7^ including carbon nanotubes,^8^ nanofiber junctions^9^ or vesicles,^10^ as well as the formation and manipulation of liquid nanodroplets.^11,12^ This enables experimental investigations into chemical reactions in nanoconfined solutions, dubbed “nanosolvation” for short.

In the context of nanosolvation, it is of paramount importance to understand the different effects playing a role in such nanoreactors which makes them a completely different medium from the bulk. For instance, theoretical works based on statistical arguments^13,14^ have coined the term “nanoconfinement entropic effects” to describe the way in which chemical equilibrium is affected when considering only a small amount of molecules, observing a considerable shift w.r.t. the equilibrium in the macroscopic limit originating in the reduction in number of reactant–product mixed microstates. Along this line, there are extensive studies on nanoconfinement effects on chemical processes. In particular, a large body of work exists on water confined in reverse micelles.^15^ These are now commonly used as templates for nanoparticle synthesis,^16^ but also reactions have been studied in these confined spaces including oligomerization,^17^ conformational equilibria^18^ and especially proton transfer.^19,20^ However, unraveling the special chemistry observed in these systems is complicated by possibly strong interactions of both reactants and water with the charged or highly polar groups of the surfactant,^19,20^ which renders it difficult to separate nanoconfinement effects from those that depend on the specific nature of the interface.

More weakly interacting confining environments like carbon nanotubes^21^ or other inert nanopores clearly reveal nanoconfinement effects on water itself. Using such systems, studies relevant to biology expose strong solvent effects due to crowding and nanoconfinement on conformational equilibria of biomolecules.^2,22–25^ Most of them explain this role in terms of entropic factors, solvent entropy in nanoconfinement being much lower than in the bulk due to the more structured character of confined water. On the other hand, other studies consider the fact of the nanoconfined solvent, itself, having properties far different from bulk water as one of the possible explanations.^26^ In particular, several studies revealed the highly inhomogeneous and anisotropic nature of the polarization fluctuations of interfacial^27–29^ and nanoconfined^21^ water, but so far no direct link has been established between these observations and the fact that some physical/chemical processes in nanoconfined water differ greatly from those in the bulk regime. Moreover, similar studies^30^ of water confined by *soft* interfaces show remarkably different results compared to what is observed in water at *hard* interfaces.^27–29^ This observation strongly indicates that the vast existing experimental knowledge about water under soft confinement conditions, using *e.g.* micelles or lipid bilayers, may be more misleading than helpful when rationalizing the behavior of water in mineral confinement.

**Fig. 1:**
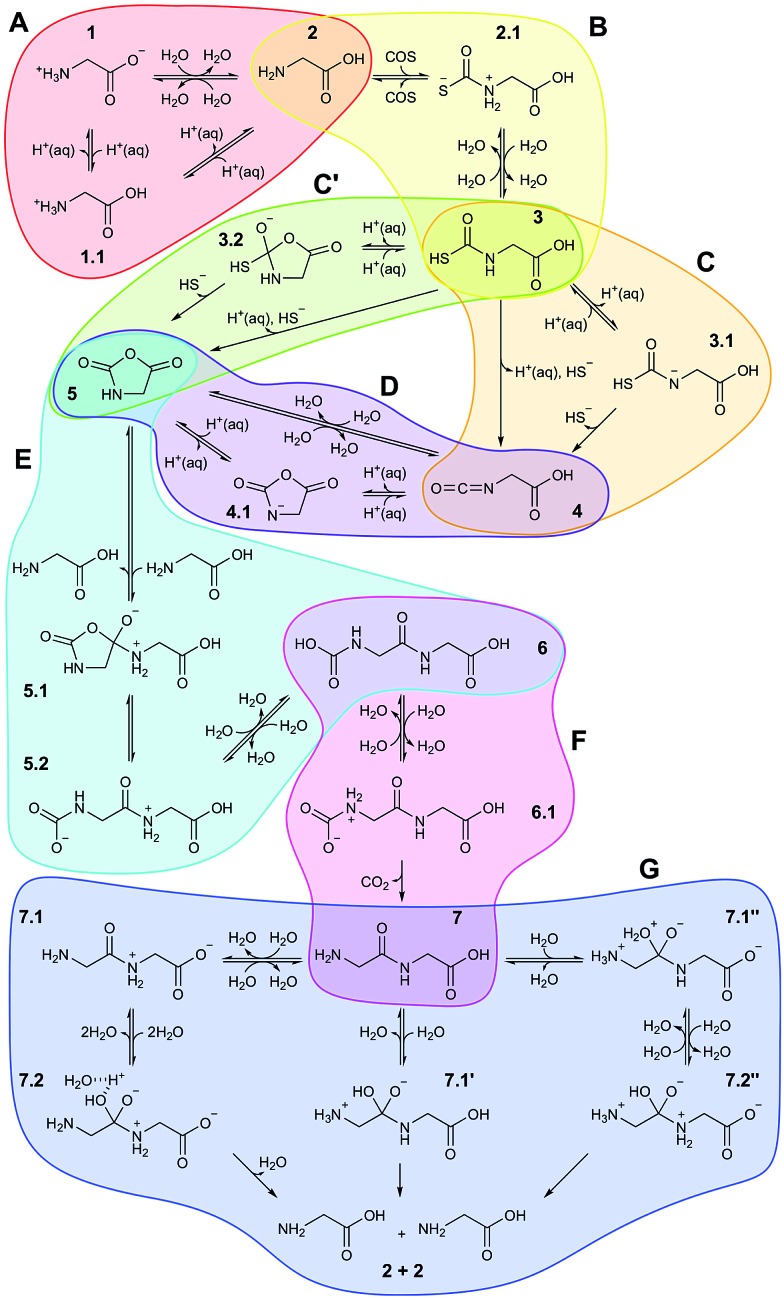
Full reaction network underlying the prebiotic peptide cycle studied in nanoconfined water in comparison to bulk water. Each colored bubble shows reaction sequences studied in a separate simulation. Reactants/products are labeled with integer numbers, while decimal numbers indicate reaction intermediates or transition states. Reactions A–D comprise the ‘activation’ part of the cycle, in which glycine is transformed in the NCA 5, while reactions E and F are the ‘elongation’ part of the cycle resulting in diglycine 7. Reaction G is the back-reaction, *i.e.* peptide hydrolysis, studied in order to evaluate peptide stability at the different conditions (see text): AMB, unprimed species; HPW, single-primed species; NCW, double-primed species. In addition to the charge state of the reactive functional groups involved in the distinct chemical reactions as indicated in the scheme, the protonation state of non-reactive ‘spectator’ groups (see text) such as carboxyl can also change depending on the conditions. These details are not included in the scheme but exhaustively analyzed in Sec. V of the ESI.†

Based on all this evidence, it becomes clear that the distinct properties of nanoconfined water must greatly impact on any wet-chemical reaction occurring therein – yet this is largely ‘terra incognita’. Here, in order to disclose how nanosolvation affects chemical processes we have studied by means of large-scale *ab initio* MD simulations^31^ an extensive set of distinct chemical reactions in a realistic setup modeling a nanoconfined water lamella between mineral (mackinawite, FeS) sheets. The particular set of reactions (Fig. 1) moreover comprises a full putative ‘prebiotic peptide cycle’ in which an amino acid (glycine) is activated (*via* addition of carbonyl sulfide, COS, and subsequent formation of its *N*-carboxyanhydride, NCA) to yield a peptide upon condensation with another amino acid (glycine). Previously, this cycle has been extensively studied by us under different conditions such as bulk water at both ambient and extreme conditions^32,33^ and in the presence of a pyrite surface.^34,35^

As opposed to pyrite, mackinawite is an iron–sulfur mineral which presents a layered structure that forms cracks and can be easily exfoliated. It is found at deep-see hydrothermal vents,^36^ where the layered cracks can be intercalated by water, thus yielding realistic nanoreactor environments for studying prebiotic chemistry in hot-pressurized water within the so-called “Iron–Sulfur World” hypothesis.^37,38^ This setup allows us to study realistic nanoconfinement effects on many reaction classes including addition, elimination, cyclization, condensation and hydrolysis reactions using chemically inert^39–41^ inorganic slit pores in contrast to nanoconfinement using reverse micelles. All these reactions are found to display remarkable differences in nanoconfined water at elevated temperature and pressure conditions compared to the corresponding bulk solvation regime. Given the fundamental nature of the aforementioned reactions, the extracted findings will be of broad importance much beyond the specific case.

## 2 Methods

### 2.1 Computational approach

**Fig. 2:**
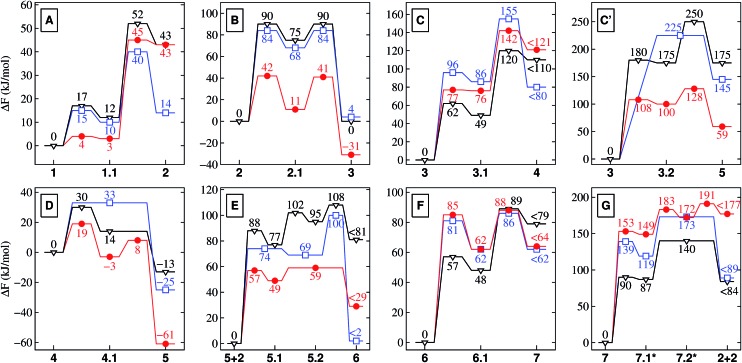
Free energy profiles for the different reactions depicted schematically in Fig. 1. These relative free energies are provided in kJ mol^–1^ for nanoconfined water at high temperature and pressure (NCW, red circles), hot-pressurized bulk water (HPW, blue squares), and bulk water at ambient conditions (AMB, black triangles). Note that *K*_B_ *T*_500_ = 4.2 kJ mol^–1^ (for NCW and HPW) and *K*_B_ *T*_300_ = 2.5 kJ mol^–1^ (for AMB). Depending on the conditions, 7.1* and 7.2* correspond to the NCW (double-primed), HPW (single-primed) or AMB (unprimed) species in Fig. 1 for which the corresponding energies are reported (in red, blue, black).

The system set-up and the methods are based on our preliminary characterization of neutral, acidic and basic water nanoconfined between mackinawite sheets.^39–41^ The PBE density functional^42^ is used, along with a plane wave cutoff of 25 Ry and ultrasoft pseudopotentials^43^ containing d-projectors for sulfur and semicore states as well as scalar relativistic corrections for iron. The *ab initio* MD simulations^31^ are carried out with the Car–Parrinello method as implemented in CPMD.^44^ The temperature of nuclei and electrons is controlled with massive Nosé–Hoover chain thermostats,^45^ with a fictitious orbital mass of 700 a.u., a timestep of 2 a.u., substituting D for H masses, and using a very high-order Suzuki–Yoshida algorithm to properly integrate these thermostat equations of motion. The systems were equilibrated for 5 ps before starting the production runs. Metadynamics in its extended Lagrangian formulation^46^ is employed in order to accelerate the reactions and to reconstruct the free energy surfaces in the space of the selected collective variables.^31^ Extensive technical details including validation of the theoretical approach, simulation protocols, error estimations, and a detailed account of all individual reactions steps depicted in Fig. 1 and 2 are provided in the ESI.†

### 2.2 Model systems

We employ the same model for water in ‘moderate nanoconfinement’ between mackinawite sheets as devised previously.^39–41^ It consists of two Fe_32_S_32_ parallel layers situated at the top and bottom of a supercell with *c* = 16.73 Å, preserving the ideal (vacuum) spacing of 5.03 Å between the neighboring layers of adjacent supercells, and a slab pore width of ∼6.7 Å in the middle of the supercell (while keeping the ideal *a* = *b* = 14.69 Å values). The top-most and bottom-most S atoms are frozen at their ideal positions. The slab pore is filled with the reactants of the specific reaction and 47H_2_O molecules for reactions A–D or 45H_2_O molecules for E–G as depicted in the snapshots, which corresponds roughly to an estimated pressure of ∼20 MPa at the fixed simulation temperature of 500 K.

## 3 Results

In preliminary studies we have characterized the properties of nanoconfined water in mackinawite,^39–41^ which established that for ‘moderate’ nanoconfinement (*i.e.* a slit pore width of ∼7 Å), water is stratified as a water bilayer around a well defined water depletion region but still features liquid-like dynamics. In stark contrast to other systems such as typical reverse micelles featuring two distinct water ensembles (‘core’ and interfacial),^47^ such nanoconfined water in mackinawite is purely interfacial water.^39–41^ Yet, this nanoconfinement has been shown to not hinder the transfer of excess protons, which takes place by Grotthuss diffusion like in the bulk regime.^40^

To set the stage, we will highlight the main aspects of the reaction cycle depicted in Fig. 1 in the sense of comparing the results in nanoconfined water at high temperature and pressure (NCW: 500 K and ∼20 MPa) to those previously obtained in the bulk solvent,^32,33^ *i.e.* in hot-pressurized bulk water (HPW: 500 K and ∼20 MPa) and in bulk water at ambient conditions (AMB: 300 K and ∼0.1 MPa), for which the free energy profiles are compiled in Fig. 2; we refer to Secs. II and III-D in the ESI† for a thorough assessment of the reliability of our theoretical approach for estimating the (free) energy differences along the reaction paths, including an assessment of PBE compared to SCS–(RI)–MP2 (ref. 48 and 49) data for the reaction classes of relevance in the present context and extensive convergence tests for the metadynamics simulations.

In the context of comparing reactions in nanoconfined spaces to reactions in reference systems, we note that studying the relevant reactions in the gas phase will not provide useful insights. This can be easily illustrated already for the first reaction step A being the glycine zwitterionic equilibrium: a glycine molecule in gas phase is neutral while the change to the zwitterionic form as preferred in aqueous environments requires the addition of several explicit water molecules,^50,51^ not to mention continuum solvation modeling that is unable to treat de/reprotonation reactions involving H-bonding solvent molecules as active ingredients. However, even the attempt to study de/reprotonation of glycine in aqueous environments using a microsolvation approach is doomed to fail because the H-bonding topology of the solvation shell has been shown recently to critically determine the conversion of neutral and zwitterionic forms because of the intimate coupling of the de/protonation reactions of the carboxyl and amino groups.^52^ This is in stark contrast to what happens in a bulk-like environment (as we indeed observe in our simulations, see below) where the de/reprotonation of these two groups is completely decoupled since the environment accepts and provides protons on demand. The particular de/reprotonation reaction A is just one striking example to illustrate our point. In conclusion, we consider such gas-phase-like calculations not to be appropriate in order to provide useful references for chemical reactions under nanoconfinement conditions, whereas our AMB and HPW systems serve this purpose as will be shown throughout the subsequent discussions.

These findings will provide the foundation for working out the general features of nanosolvation effects in the Discussion section, whereas the rich details of the individual reaction steps are elaborated in the ESI.† Note that in all reactions in NCW (Fig. 1) the stratified structure of the water lamella as described earlier^39–41^ is retained. Another common aspect in all of them is the absence of chemical interactions between both, solvent and solute molecules and the confining mineral sheets (as illustrated in Fig. 6, 8 and 9 in the ESI†).

### 3.1 Stability of charged and charge-separated species

**Fig. 3:**
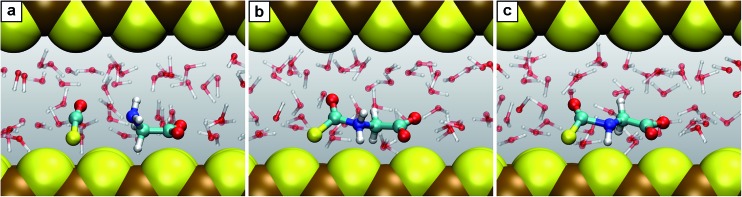
Snapshots for the reaction B. (a) Glycine together with carbonyl sulfide (2 + COS); (b) intermediate 2.1; (c) product *N*-thiocarboxyl glycine 3. Color code (used for all such snapshots): carbon (cyan), nitrogen (blue), oxygen (red), hydrogen (white), sulfur (yellow) and iron (brown). The two independent mackinawite slabs, which confine the water pore being the water-filled reaction space, are shown as van der Waals spheres. The reacting species are depicted as large balls-and-sticks whereas the solvation water molecules are represented as transparent balls-and-sticks.

**Fig. 4:**
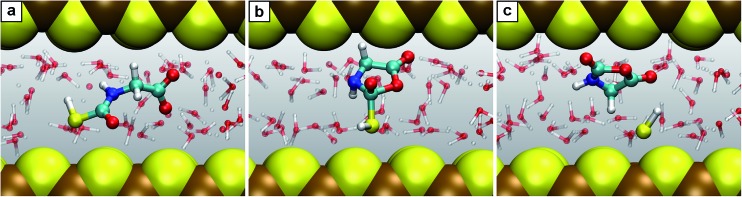
Snapshots for the reaction C′. (a) *N*-Thiocarboxyl glycine 3; (b) intermediate 3.2; (c) product *N*-carboxyanhydride NCA 5 with the SH^–^ leaving group.

Nanoconfinement is found to generically facilitate the formation of charged or charge-separated species taking part in chemical reactions which in most cases is revealed by comparing the free energy profiles for NCW and HPW conditions (Fig. 2). To start with, in the glycine zwitterionic equilibrium A, the zwitterionic form 1 is found to be much more stable in NCW than the neutral one 2, unlike in HPW (but quite similar to AMB). In reaction B (Fig. 3), the relative stability of the charged intermediate 2.1 in NCW, measured as the free energy barrier for reverting either to reactant 2 or product 3, is twice that in HPW (32 *vs.* 16 kJ mol^–1^). In step C, the barrier for the deprotonation of neutral species 3 to yield charged 3.1 is lower in NCW than in HPW. In reactions C′ (Fig. 4) and D, the charged species 3.2 and 4.1 can be identified as stable intermediates in NCW, while in HPW they were observed only as transition states. Consequently, nanoconfinement induces here a qualitative change in reaction mechanisms from concerted in HPW to stepwise in NCW thus involving charged species. Also in E, the charged adduct 5.1 is more stable in NCW than in HPW, while the diglycine hydrolysis G proceeds stepwise through the charged intermediates 7.1′′ and 7.2′′. On top of these general trends, an important specific observation not reflected in most of the free energy profiles concerns all those species that feature a carboxyl group that is not directly involved in the chemistry of the particular reaction step (except for A where its protonation equilibrium is explicitly studied). Changes of the protonation state of these ‘spectator’ functional groups, and thus changes of the charge state of solvated species, are exhaustively investigated in Sec. V of the ESI† and further confirm the charge-stabilization trends in NCW that were disclosed above when analyzing the respective free energy profiles. Being typically weak acids, these non-reactive carboxyl groups are consistently found in the neutral –COOH form in HPW, while in NCW they can be either essentially fully deprotonated and thus in the anionic –COO^–^ charged state or in equilibrium with the neutral state, much like what is known from ambient bulk water, AMB.

### 3.2 Addition *vs.* elimination reactions and small *vs.* big reactants

As it is readily seen in the free energy profiles (Fig. 2), for some reactions the free energy barriers in NCW are dramatically lower (by up to ≈ 50%) than in HPW, while for others the barriers are only moderately lower or are even higher – why so? In the first class we find addition reactions (B and E) and intramolecular cyclizations (C′ and D), whereas in the second one there are elimination reactions (C and F) and the special case of reaction G consisting in a water addition followed by dissociation of the dipeptide. Moreover, another trend observed throughout the reaction cycle is that the largest quantitative differences between the profiles in NCW and HPW are found for reactions that involve small reactants, while these profiles become more similar when larger species are involved. There is even a reversal found for reaction G, where the overall free energy barrier is now higher in NCW compared to HPW.

### 3.3 The solvent as an active player

**Fig. 5:**
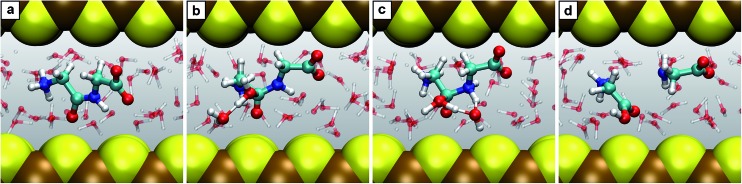
Snapshots for the reaction G. (a) Diglycine 7; (b) intermediate 7.1′′; (c) formation of the intermediate 7.2′′; (d) two glycine molecules being the products of hydrolysis. For the intermediate 7.1′′ the particular solvent water molecule is highlighted which contributes to stabilizing the excess proton of the attacking H_2_O. In case of formation of the intermediate 7.2′′, that solvent H_2_O is highlighted which donates at the same time a proton to the nitrogen and accepts the excess proton from the attacking H_2_O.

Reaction G presents different mechanisms for all the studied conditions. In particular, the role of the solvent is quite different comparing HPW and NCW. The first step in HPW is the attack of a water molecule on the carbon atom of the peptide bond with a concerted proton transfer to the terminal amino group, which is followed by a proton donation of this same amino group to the nitrogen atom of the peptide bond (Fig. 1: steps 7 ⇄ 7.1′ → 2 + 2). The solvent does not participate in the reaction other than contributing that water molecule which hydrolyzes the dipeptide. This is distinctly different from the mechanism revealed in NCW (Fig. 1 and 5). Here, after the water attacks the carbon of the peptide bond to form 7.1′′, the excess proton at the bound water is not immediately released. It rather stays loosely attached to the OH group, which shares it with one of the surrounding waters or with an oxygen of the terminal carboxyl group in a similar fashion to the shared proton in a so-called Zundel complex (see *e.g.* Fig. 1 in ref. 40). In the second step, deprotonation of the attacking water and protonation of the peptide's N atom to form 7.2′′ was observed through two reaction channels, both of them involving Grotthuss-like proton diffusion through solvation water.^40^ Incidentally, at AMB conditions, the solvent was also seen to act as an active proton donor/acceptor rather than merely acting as a ‘water supply’ as in HPW, despite the mechanism in AMB (Fig. 1: steps 7 ⇄ 7.1 ⇄ 7.2 → 2 + 2) being different from either HPW or NCW.

## 4 Discussion

The stage is now set to appreciate the salient effects due to nanoconfinement. The first immediate result of our investigation is the observation of charged and charge-separated species being stabilized in NCW with respect to the corresponding bulk regime, HPW, which leads to the aforementioned *qualitative* changes in reaction mechanisms. The key to understanding is based on the observation that in many instances the mechanism in *hot-pressurized but nanoconfined* water, NCW, is more similar to the one in *ambient bulk* water, AMB, rather than to hot-pressurized bulk water, HPW. This is especially clear in reactions C′ and D, which are stepwise in both NCW and AMB but concerted in HPW. Moreover, the different protonation state of the carboxylic spectator groups in NCW or AMB conditions compared to HPW (*i.e.* mostly anionic *vs.* neutral, respectively) follows and thus supports this general trend as specifically disclosed in Sec. V of the ESI.† Another important finding is that the mechanistic involvement of solvent in reaction G in NCW is more similar to AMB than to HPW. In HPW, the only role played by the solvent is to provide the particular water molecule which hydrolyzes the dipeptide, whereas all proton transfers are intramolecular and thus not solvent-mediated processes which avoids charge separation. In both AMB and NCW, the opposite is true: here solvent molecules become reactants since they act as active proton acceptor/donor species, thereby promoting the formation of charged intermediates. In previous studies^32,33^ the differences between the mechanisms in AMB and HPW were attributed to the change in the dielectric properties of bulk water. It is well established that the dielectric constant of pure bulk water gets significantly reduced^53,54^ upon raising temperature and pressure from ambient to extreme conditions. The reduction is by more than a factor of two in the present case (from *ε* ≈ 80 at AMB to roughly 30 in HPW), which essentially makes HPW another solvent than at ambient conditions. Thus, charged or charge-separated species are much favored in ambient conditions, while extreme conditions discourage the formation of these and stabilize the neutral form of reactants and intermediates in the bulk. In fact, the peculiar chemistry taking place in hot bulk water is usually explained^53^ in terms of its reduced dielectric constant.

In distinct contrast to what has been observed earlier in the limit of bulk solvation, our results show that *charged* species are again *stabilized* in NCW – which therefore must be caused by nanoconfinement as such. Unfortunately, in several of the studied reactions this effect is at interplay with other effects, being mainly of a purely geometric/steric nature as will be discussed below. Given the breadth of distinct chemical reactions studied, the resulting complexity makes it unfortunately impossible to strictly quantitatively compute the corresponding energetic stabilization contributions of charged species in NCW including, in particular, the crucial transition states. We can, however, draw qualitative conclusions based on recent studies of interfacial effects on pure water that allow us to understand general trends that we broadly observe as a result of nanoconfinement.

It has been repeatedly shown that the parallel component of the dielectric tensor of water at hard interfaces at ambient conditions increases significantly when approaching the interfacial region from the bulk.^27–29,55^ This phenomenon even induces what is called dielectric superpermittivity of water confined within carbon nanotubes.^21^ Moreover, it can be analytically shown that the average polarization fluctuations (which are directly proportional to the usual static dielectric constant *ε* in the limit of bulk solvation according to eqn (4.8) in the ESI†) are dominated right at the interface by the unusually high value of the parallel component of the dielectric tensor of interfacial water,
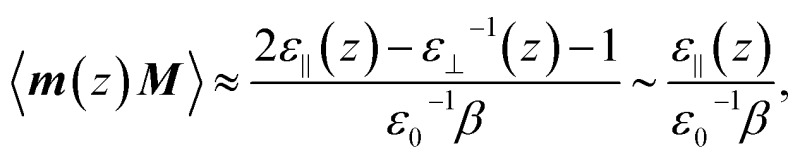
since *ε*_∥_(*z*) increases steeply from ≈70 far from the interface to roughly 150/120 at hydrophobic/hydrophilic interfaces^27,28^ while *ε*_⊥_^–1^(*z*) varies only from about –1 to +1 upon approaching the interface (see Sec. IV in the ESI† for derivation and notation). The simplified relation valid close to the interface suggests that interfacial water, such as NCW, could host charged or strongly polarized species much better than bulk water, such as HPW, can; it is noted in passing that this effect could be even more pronounced for water confined inside nanotubes.^21^ Unfortunately, a rigorous formalism to compute the energetic stabilization of charged molecular species in water directly from the polarization fluctuations is so far only available for way too simple cases such as spherical ions in bulk water^56^ (see Sec. IV in ESI†).

A complementary perspective might be offered, at first sight, by considering the stratified nature of water in the slit pore with its *locally* enhanced density close to the confining walls.^39–41^ This could be considered to shift the system at constant temperature into another region of state points in an appropriately defined phase diagram of nanoconfined water. Yet, apart from non-trivial complications, the required increase of density or pressure is known to increase the dielectric constant in the limit of homogeneous liquid bulk water,^57^ which connects with the previous argument about enhanced polarization fluctuations at the interface as a possible origin of the charge-stabilization in NCW. Finally, we note that it has been recently shown^30^ that water confined by soft interfaces shows only a very small enhancement of *ε*_∥_, in stark contrast to the strikingly pronounced enhancement of *ε*_∥_ that has been demonstrated repeatedly for water at hard interfaces.^27–29,55^ This clearly suggests that water confined by hard surfaces, such as mackinawite sheets in the present case, possesses distinctly different properties from water confined in soft media. This implies not only that the extensive knowledge of (nano)confined water in media such as reverse micelles or lipid bilayers does not apply to water lamellae within mineral sheets, but also that further studies must be performed upon varying the material of the confining surfaces in order to understand the role and influence of the confining media on the properties of water hosted therein.

We must remark, however, that the charge-stabilization effect in NCW compared to HPW – regardless of its origin – is a direct observation based on our simulation results and is, moreover, consistently observed in all reactions in the same way as it is observed when comparing AMB to HPW. In some of the reactions, this is clearly realized when comparing the free energy profiles. A clear example is the zwitterionic *vs.* neutral equilibrium A of glycine: the neutral form 2 is seen to be stabilized upon raising temperature and pressure from AMB to HPW, whereas nanoconfinement destabilizes 2 greatly w.r.t. the zwitterionic species 1. In other showcases, such as for species 3.2 and 4.1 (being stabilized intermediates in NCW but high-energy transition states in HPW, see Fig. 2), this even results in a different mechanism for the same reaction depending on the conditions, *i.e.* stepwise in both NCW and AMB *vs.* concerted in HPW.

In contrast, in all those reactions where the free energy profiles for two different conditions are unexpectedly similar, for instance as encountered when directly comparing AMB to HPW in reaction B or HPW *vs.* NCW for reaction F, the protonation state of the non-reactive spectator group is *different* at the two conditions compared (being always neutral at HPW *vs.* preferentially anionic at AMB or NCW). In HPW, the spectator group can stabilize an otherwise negatively charged species by becoming protonated, thus producing an overall neutral species, while it remains deprotonated at AMB (or NCW) conditions, which readily explains unexpected features of the distinct free energy profiles of reaction B (respectively F) after considering the protonation states of species 2, 2.1 and 3 with the help of Fig. 7 in the ESI† (respectively of species 6, 6.1 and 7 in Fig. 11 in the ESI†); see Sec. V in the ESI† for a comprehensive discussion of all cases. Thus, non-reactive spectator groups can act like ‘buffers’ that minimize the impact of the changing conditions on the reacting charged groups (being directly involved in the ongoing chemistry) by changing the *overall* charge state of the reacting species independently from the ongoing reaction.

We conclude at this stage that all these details qualitatively confirm the general trend of an increased ability of NCW to stabilize charged or charge-separated (zwitterionic) species compared to HPW, as well as the associated changes of reaction mechanism, as a result of nanoconfinement.

Another key observation regards the two major trends revealed in the Results section upon comparing the free energy profiles in NCW *vs.* HPW. Firstly, the barriers in addition reactions (either inter- or intramolecular) are considerably lowered in NCW, while in elimination reactions they are only moderately lower or even higher. Secondly, the differences between NCW and HPW are less pronounced in reactions that involve bulky molecules than in those of smaller molecules. These two trends have a clear steric origin: in the case of intermolecular addition reactions, the two-dimensional nanoconfinement imposed by the mineral layers restricts the diffusion of the solvated molecules, thus favoring reactive encounters of the reactants compared to the bulk environment once they are close. Such is the case of reaction B, where analyses of the trajectories reveal that the reactants, glycine and COS, always reside in the same water layer. Roughly speaking, this leaves only two translational degrees of freedom for the relative position of one reactant relative to the other, as opposed to the three degrees of freedom in the bulk, thus leading to the pronounced changes observed in the free energies. A similar argument applies to the cyclizations C′ and D. The confining environment restricts the conformational landscape of these molecules: given their size, the fully stretched conformations are only possible if the molecule lies parallel or slightly inclined w.r.t. the mineral surface while folded conformations are possible regardless of the orientation (Fig. 4). This helps the reactants in adopting the necessary conformation for the formation of compact cyclic intermediates such as 3.2 (nicely visualized in Fig. 4) and 4.1, which therefore favors the corresponding reactions under NCW conditions. On the other hand, elimination reactions like C and F do not benefit from this dimensionality reduction, thus the smaller differences found for them between the NCW and HPW profiles.

**Fig. 6:**
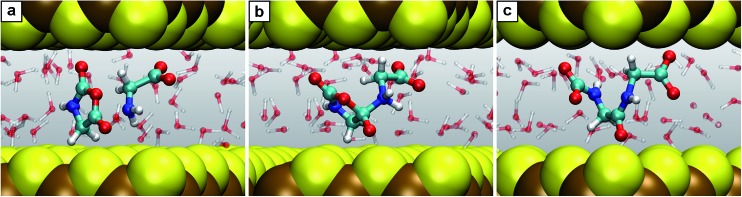
Snapshots for the reaction E. (a) Glycine together with *N*-carboxyanhydride (2 + 5); (b) intermediate 5.1; (c) product *N*-carboxyldiglycine 6.

The second trend has its origin in the steric hindrance coming into play in the reactions where bigger molecules are involved. Take for instance reactions B and E. In the first one, a glycine molecule reacts with a compact molecule, COS. Being a linear molecule, the C atom can be attacked by glycine's amino group from any angle except those at the ends of its axis. In contrast, in reaction E, glycine must attack the C2 atom of the NCA, a planar molecule of larger size. In this case, the attack is only possible along orientations roughly perpendicular to the plane of the heterocycle. Imposing nanoconfinement on B greatly facilitates this reaction (lowering the barrier in NCW by 50% w.r.t. HPW): while the diffusive modes are restricted by the mineral surfaces as previously explained, the small size leads to reactive encounters (Fig. 3). In contrast, for reaction E, there is a balance between the advantage of the molecules being pushed together and the disadvantage of the confining environment that hinders re-orientations of the reactants (Fig. 6), which could result in shielding the molecule's reactive sites. While the former facilitates the reaction in NCW w.r.t. HPW, the latter counteracts thus resulting in an only about 23% decrease of the overall barrier, in contrast to the huge 50% decrease of reaction B where no such hindrance exists. Similar steric influences are observed for reaction G, where a water molecule must attack an atom at the center of the dipeptide, which is more shielded (due to the confining surfaces in conjunction with closeby parts of the chain) compared to the terminal atoms which are involved in other reactions. It is noted that it is precisely this protection against water attack what renders peptide hydrolysis in NCW unfavorable with respect to HPW conditions, since the subsequent elementary steps are actually favored in NCW w.r.t. HPW (compare in Fig. 2 the relative barrier in NCW for going from 7.1′′ to 2 + 2, *i.e.* 42 kJ mol^–1^, *vs.* that barrier in HPW for 7.1′ to yield 2 + 2, *i.e.* 54 kJ mol^–1^).

Coming now back to the prebiotic peptide cycle, the dramatic drop in the free energy barrier for the key activation reaction C′ in NCW makes this simple process preferred over the indirect pathway C plus D. Thus, nanosolvation favors amino acid activation *via* direct cyclization over the most indirect isocyanate route (reactions C and D), which is preferred in AMB and HPW conditions. In addition, taking into account that the highest free energy barrier for the entire peptide formation process is significantly lowered in NCW, it is clear that nanoconfinement favors peptide formation, while peptide hydrolysis (*i.e.* reaction G) is hindered concurrently. Regarding further elongation of the dipeptide 7, this would take place *via* reaction of another NCA with the easily accessible *terminal* amino group of the dipeptide, whereas peptide hydrolysis will be sterically hindered since it requires water attack at the buried peptide bond. In consequence, polypeptide formation in aqueous conditions should be favored in NCW over *both* HPW and AMB conditions, *i.e.* nanosolvation favors the synthesis of peptides in aqueous environments.

This result along with our general conclusions will not only be of immediate interest to synthetic chemistry, but may have deep implications for prebiotic chemistry. While it is certainly out of the scope of this purely computational investigation into reaction mechanisms to contribute to origin-of-life research, it is interesting to see that layered minerals, like mackinawite or fougèrite, have been considered to provide habitats for the emergence of local metabolisms.^58,59^ In this context, it has been suggested that the water-filled interlayer nanometric channels offered by these minerals could not only serve to establish proton and electron gradients leading to the emergence of what has been called a ‘pyrophosphate synthetase nanoengine’^58^ (*cf.* Fig. 5 in ref. 59), but also as inorganic templates which would have facilitated the formation of peptide α-sheets and even amyloids^58^ (a speculation directly backed up by our present results for the peptide cycle), which in turn could have become protoenzymes by sequestering metals or inorganic clusters. We therefore emphasize that the unveiled peculiar chemistry taking place in nanoconfined water might provide new clues for better understanding the function of primordial inorganic membranes comprised of iron- and sulfur-rich layered precipitates^36^ which have been put forward to be precursors of molecular machines such as pyrophosphate synthetase.^58,59^

## 5 Conclusions

In summary, the investigated set of reactions in nanoconfined water at high temperatures and pressures features pronounced differences in energetics and mechanisms with respect to bulk water at the same conditions. These can be traced back to a unique combination of factors, namely the different charge-stabilizing ability of interfacial water w.r.t. the bulk as well as steric factors intrinsic to nanoconfinement which make nanoconfined water in slit pores offered by layered minerals a whole new medium for chemical synthesis. The presented prebiotic peptide cycle is a good example of the way in which nanoconfined water as a solvent opens up an entirely new free energy landscape for exploring novel synthesis routes. While changing the thermodynamic conditions from ambient to hot-pressurized bulk water greatly reduced free energy barriers, at the same time the formation of charged intermediates was penalized due to unfavorable dielectric properties. In nanoconfined water at extreme conditions, in contrast, it is possible to achieve significant thermal activation and to concurrently favor reactions involving charged species. Clearly, the key phenomena and mechanistic concepts unraveled here for chemical reactions in nanoconfined solvent not only apply to the specific reactions investigated herein, but are of fundamental importance to chemistry in nanoconfined water as such.
