# Supplementary material for: Chemistry in nanoconfined water
Source: Chem Sci. 2017 Mar 20;8(5):3444–52. doi: 10.1039/c6sc04989c (PMC5418629; doi:10.1039/c6sc04989c)
Supplement: Supplementary file 1 [file SC-008-C6SC04989C-s001.pdf]

Electronic Supplementary Material (ESI) for Chemical Science.
This journal is © The Royal Society of Chemistry 2017

Electronic Supplementary Information for

Chemistry in Nanoconfined Water

Daniel Muñoz-Santiburcio and Dominik Marx

Lehrstuhl für Theoretische Chemie, Ruhr–Universität Bochum, 44780 Bochum, Germany

I. GENERAL INFORMATION

The basic computational approach and system setup employed in this study is the same as used in our preliminary characterization of neutral, acidic and basic water lamellae confined between mackinawite (FeS) sheets [1–3]; the technical details are included in Section II for completeness. The reactions comprising the prebiotic peptide cycle have been previously studied by us in bulk water at ambient and hot–pressurized conditions [4, 5], which serve as our (AMB and HPW) references to study nanoconfinement effects (using NCW conditions) being the focus of the present investigation. The ab initio metadynamics technique is used for accelerating the reactions and for computing the corresponding free energy surfaces (FES) as reviewed in Section III where also the specific reaction coordinates and corresponding parameters used in the present case are compiled. Therein, detailed convergence tests and error estimates for the free energy profiles are provided. In Section IV, we analyze the dielectric properties of interfacial water in terms of its polarization fluctuations on the basis of recent results [6, 7]. Last but not least, we expose in detail the mechanistic aspects for each one of the studied reaction steps separately in Section V.

II. AB INITIO MOLECULAR DYNAMICS

All calculations were performed within spin–restricted Kohn–Sham density functional theory in its plane wave / pseudopotential formulation [8]. The PBE [9] exchange–correlation functional was chosen and the core electrons were taken into account using Vanderbilt's ultrasoft pseudopotentials [10] containing additional d–projectors in case of sulfur as well as scalar relativistic corrections and semicore states for iron. This particular approach has been demonstrated to yield an accurate description of the electronic structure of the system of interest [11, 12]. In the context of van der Waals corrections, it is important to note that the plain PBE functional used here is robust and yields accurate results for a wide range of systems including liquids, solids and molecules *without* adding a dispersion correction [13]. Moreover, for hydrogen-bonded systems in particular, the inclusion of well-established dispersion corrections is not at all recommended since it *increases the average error* significantly (see left panel of Fig. 8 in Ref. 13) rather than improving the energetics. In addition, the plain PBE functional has been shown to reproduce the dielectric constant

of water at both ambient [14] and supercritical conditions [15] with remarkable accuracy, which is a significant advantage of this functional for the present study.

Finally, we have assessed the reliability of PBE for estimating the (free) energy differences along the studied reactions by a suitable comparison with the SCS–(RI)–MP2 [16, 17] method, which performs considerably better than plain MP2 for non–covalent interactions [18]. For the two representative reactions **A** and **C'** (probing respectively de/protonation equilibria and covalent bond formation being key reaction classes along the studied peptide cycle) at HPW conditions, we sampled representative snapshot configurations along these reaction pathways (close to the reactant, transition, and product states that characterize the entire reaction profiles) as obtained from the trajectories of the metadynamics simulations. By eliminating the most distant water molecules to the solutes, we obtained the corresponding 'microsolvated configurations' for each species (being overall charge neutral), which are amenable to quantum chemical MP2 calculations. We performed single-point energy calculations with these structures employing both PBE and SCS–(RI)–MP2 with the TZVPP basis set [19] as implemented in `Turbomole` [20]. By comparing the energy of the different species relative to the reactant state of each reaction, shown in Fig. 1, it is clearly realized that PBE is indeed able to faithfully predict the underlying relative energies along the reaction pathways from reactant to transition to product states.

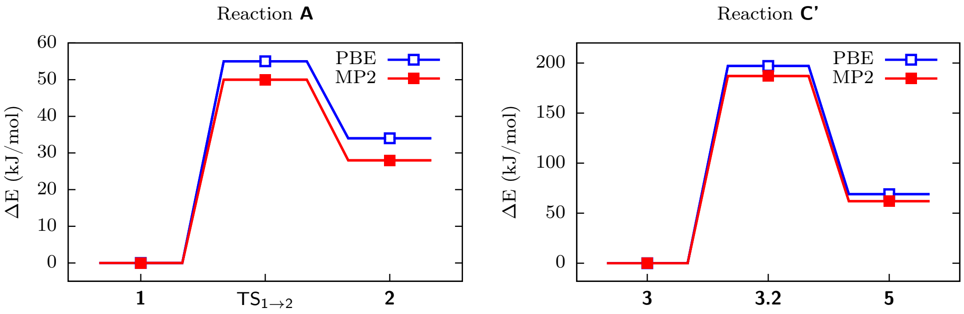

Figure 1: Energy difference ΔE relative to the reactant state, being **1** for reaction step **A** and **3** for reaction **C'** according to Figure 1 in the main text, computed with both the PBE and SCS-MP2 methods. The corresponding structures are microsolvated with 20 H_2O molecules in reaction **A** and 22 H_2O in reaction **C'** as given by the configuration snapshot sampled from the respective metadynamics simulations. See text for details.

The ab initio molecular dynamics (AIMD) simulations were performed using the Car–

Parrinello propagation scheme [21] along with massive Nosé–Hoover chain thermostats [22] for nuclei and electronic orbitals, using a chain length of 8 and a high-order Suzuki–Yoshida algorithm to properly integrate the equations of motion of the thermostats. A molecular dynamics time step of $\Delta t = 0.0484\,\text{fs}$ was used for the integration of the Car–Parrinello equations of motion, the fictitious mass for the orbitals was 700 a.u., and as usual the hydrogen masses were substituted by deuterium masses for technical reasons. All simulations have been performed with the `CPMD` simulation package [23].

There has been some debate in the literature as to the impact of the fictitious mass parameter on Car–Parrinello simulations, and it is sometimes believed that using somewhat higher fictitious masses may lead to artifacts in non–dynamical properties such as radial distribution functions and thermodynamics, and hence in wrong hydrogen-bonding properties. These concerns have been properly answered in several studies and we refer to Section 2.4.9 in Ref. 8 for a concise discussion of this issue. Moreover, our specific simulation parameter setup (in the framework of using separate sets of thermostats for nuclei and orbitals in conjunction with using massive thermostatting of the nuclei) was thoroughly tested in our previous studies of neutral, acidic and basic water confined between mackinawite sheets [1–3]. In particular, it has been checked in Ref. 2 that Car–Parrinello propagation is stable for our system and that similarly stable propagation can be observed in test runs with both lower and *higher* fictitious masses of 500 and 900 a.u., respectively. In that same study, the structural features of the hydrated excess proton were carefully analyzed, with special focus on the limiting structures known as 'Zundel' and 'Eigen' complexes, and the radial distribution functions matched those described in earlier works [24]. It is therefore concluded that the chosen fictitious orbital mass does not artificially affect the non–dynamical properties that are computed in the present study.

III. AB INITIO METADYNAMICS

A. Theoretical Background

The metadynamics sampling technique is used to accelerate chemical reactions and to explore their reaction mechanisms by investigating the topology of their free energy hypersurfaces as obtained by this method. The essence of this technique [25] is a coarse–grained

description of the underlying chemistry by choosing a greatly reduced set $\{S_\alpha(\mathbf{R})\}$ of collective variables (CV), which are generalized coordinates depending in a very general way on the cartesian coordinates $\mathbf{R}$ of the nuclei, in conjunction with a non–Markovian (pseudo–) time evolution as reviewed in Refs. 8, 26. Here, we use the efficient extended Lagrangian formulation [27] of the metadynamics technique in which a set of auxiliary degrees of freedom $\{s_\alpha\}$ associated to the CVs is introduced.

These auxiliary variables $\mathbf{s}$ are coupled to the CVs through a simple harmonic restraining potential $\sum_{\alpha=1}^{N_S} k_\alpha [S_\alpha(\mathbf{R}) - s_\alpha]^2$, where k_α is the coupling constant and N_S is the number of CVs and thus the dimensionality of the spanned subspace. Each auxiliary variable has a fictitious mass μ_α associated to define the conjugate fictitious momenta. Within the space spanned by all CVs, a multivariate Gaussian–type potential $V^{\mathrm{MTD}}(t, \mathbf{s})$ is incremented slowly on discrete points along the trajectory of the auxiliary variables as time progresses. Here, this potential is defined as

$$V^{\mathrm{MTD}}(t, \mathbf{s}) = \sum_{t_i < t} H(t_i) \exp\left\{ -\frac{[\mathbf{s}(t) - \mathbf{s}(t_i)]^2}{2\left[\mathbf{w}(t_i)\delta s(t_i)\right]^2} \right\}, \qquad (3.1)$$

where the sum runs over all the previous metadynamics steps Δt^{MTD}, $2\Delta t^{\mathrm{MTD}}$, ..., t_i, ... with corresponding Gaussian centers $\mathbf{s}(t_i)$ where $\Delta t^{\mathrm{MTD}} \gg \Delta t$. The parameters $H(t_i)$ and $\delta s(t_i)$ determine the height and the width of the Gaussian dropped at a time t_i, respectively. To sample the available CV space efficiently, $H(t_i)$ and $\delta s(t_i)$ are adapted during the simulation according to the depth and width of the well and the progress of filling. Nonspherical free energy basins are filled efficiently by the usage of anisotropic scaling factors $\mathbf{w} = \{w_\alpha\}$ that are determined as explained in Section III C. The metadynamics time step Δt^{MTD} is an integer multiple of the AIMD time step Δt and is calculated adaptively according to the diffusion of the CVs to be described in Section III C.

The accumulated history–dependent potential $V^{\mathrm{MTD}}(t, \mathbf{s})$ slowly fills the underlying free energy surface and drives the system to other minima along the minimum free energy pathway. Due to this feature, the metadynamics technique has the capability to explore surprisingly new structures and reaction pathways (see e.g. Ref. 12). Moreover, if all proper CVs are included, i.e. if the subspace is sufficient to host the exact but unknown reaction coordinate, it provides an unbiased estimate of the free energy if carefully filled with Gaussians. Utilizing the useful relationship [28] that the negative of the accumulated biasing potential

is a quantitative estimate of the (Helmholtz) free energy

$$F(\mathbf{s}) = -\lim_{t\to\infty} V^{\mathrm{MTD}}(t, \mathbf{s}) + \text{constant} \tag{3.2}$$

in the subspace spanned by $\{s_\alpha\}$, free energy differences and free energy barriers can be obtained directly from metadynamics simulations for the explored reaction mechanism without applying any re-weighting procedure.

Such NVT free energy surfaces were determined separately for each reaction **A**, **B**, ... studied. This procedure is the analog of the standard approach used in static quantum–chemical investigations of chemical reaction sequences in the gas phase (or equivalently in continuum solvents) where (i) total energies of reactants, transition states, and products are computed separately for all individual reaction steps followed by (ii) estimating the relative free energies by invoking the harmonic approximation to compute harmonic frequencies used to correct for finite–temperature and pressure effects. Here, this well–established approach is generalized to fully dynamical ab initio simulations that do include finite–temperatures and pressures as well as solvent effects explicitly when computing relative free energies and activation barriers. In order to further improve the efficiency of the metadynamics simulations when sampling multidimensional free energy surfaces, we used the "multiple walker" algorithm [29]. Several replica of the system ("walkers") are run in parallel and each one of them contributes independently to fill the underlying free energy surface by adding Gaussians while moving on the sum of all the Gaussians added by all walkers. The scaling of this algorithm is linear with the number of walkers [30] which allows one to use a very large number of processors most efficiently in a massively parallel fashion taking advantage of platforms like the 28-rack Blue–Gene/Q system JUQUEEN at the Jülich Supercomputing Centre [31].

B. Collective Variables

In the presented work three different types of CVs $\{S_\alpha(\mathbf{R})\}$ are used for metadynamics simulations. The simplest one used is just the distance

$$d\,[\mathrm{A}-\mathrm{B}] = |\mathbf{R}_\mathrm{A} - \mathbf{R}_\mathrm{B}| \tag{3.3}$$

between two chosen atoms A and B.

The second type is the coordination number [32] between an atom A with respect to a set of other atoms B, $c\,[\mathrm{A}-\mathrm{B}]$, consisting of the same species, which is defined as

$$c\,[\mathrm{A}-\mathrm{B}] = \sum_{I\in\mathrm{B}} \frac{1-\left(R_{\mathrm{A}I}/R^0_{\mathrm{AB}}\right)^6}{1-\left(R_{\mathrm{A}I}/R^0_{\mathrm{AB}}\right)^{12}} \quad . \tag{3.4}$$

Here, $R_{\mathrm{A}I}$ is the distance between atom A and an atom I and R^0_{AB} is a fixed cutoff parameter that characterizes the typical bond distance between A and B. For each pair of atoms belonging to A and B, the function $c\,[\mathrm{A}-\mathrm{B}]$ is close to unity within the bond distance, i.e. if $R_{\mathrm{A}I} < R^0_{\mathrm{AB}}$ and approaches zero rapidly when $R_{\mathrm{A}I} > R^0_{\mathrm{AB}}$.

The third type of CV is a total coordination number $c^{\mathrm{tot}}\,[\mathrm{A}-\mathrm{B}]$ between two sets of atoms A and B, defined as

$$c^{\mathrm{tot}}\,[\mathrm{A}-\mathrm{B}] = \sum_{J\in\mathrm{A}}\sum_{I\in\mathrm{B}} \frac{1-\left(R_{II}/R^0_{\mathrm{AB}}\right)^6}{1-\left(R_{IJ}/R^0_{\mathrm{AB}}\right)^{12}} \quad . \tag{3.5}$$

The cutoff distances R^0_{AB} in Equations (3.4) and (3.5) for different pairs of species A and B used in this work are given in Table I. The particular sets of CVs utilized in the metadynamics simulations of the individual reaction steps are listed in Table II.

A – B	R^0_{AB}
N – H	1.4
O – H	1.4
S – H	1.8
C – O	1.8
C – S	2.3

Table I: Distance cutoff parameters R^0_{AB} (Å) as used in Equations (3.4) and (3.5) for different pairs of species A and B.

C. Parameters and Simulation Protocol

Our standard simulation protocol starts with equilibration runs (i.e. using regular AIMD) of $\sim$ 5 ps duration. Then, the multiple walker algorithm is initialized and several walkers

are created, starting from the same point on the FES but with random velocities for the auxiliary variables of the different replica. Before starting the deposition of Gaussians, the

Reaction	Collective Variable	Wall acting for CV values
**A**	$c\,[\mathrm{N} - \mathrm{H_{na}}]$	
	$c^{\mathrm{tot}}\,[\mathrm{O_{COOH}} - \mathrm{H_{na}}]$	
**B**	$d\,[\mathrm{N_{Gly}} - \mathrm{C_{COS}}]$	≥ 4.2
	$c\,[\mathrm{N_{Gly}} - \mathrm{H_{na}}]$	≥ 2.4
**C**	$c\,[\mathrm{N} - \mathrm{H_{na}}]$	≥ 1.3
	$c\,[\mathrm{S} - \mathrm{H_{na}}]$	
	$c\,[\mathrm{S} - \mathrm{C}]$	
**C′**	$d\,[\mathrm{O_{COOH}} - \mathrm{C_{COSH}}]$	≥ 4.5 Å
	$d\,[\mathrm{S_{COSH}} - \mathrm{C_{COSH}}]$	≥ 4.5 Å
**D**	$d\,[\mathrm{C_{iso}} - \mathrm{O_{COOH}}]$	≥ 4.3 Å
	$c\,[\mathrm{N} - \mathrm{H_{na}}]$	≥ 1.4
**E**	$d\,[\mathrm{N_{Gly}} - \mathrm{C5_{NCA}}]$	≥ 5.0 Å
	$d\,[\mathrm{O1_{NCA}} - \mathrm{C5_{NCA}}]$	
	$c\,[\mathrm{N_{Gly}} - \mathrm{H_{na}}]$	≥ 2.2
**F**	$d\,[\mathrm{N} - \mathrm{C}]$	≥ 4.0 Å
	$c\,[\mathrm{N} - \mathrm{H_{na}}]$	
	$c^{\mathrm{tot}}\,[\mathrm{O} - \mathrm{H_{na}}]$	
**G**	$d\,[\mathrm{N_{pept}} - \mathrm{C_{pept}}]$	≥ 4.0 Å
	$c\,[\mathrm{N_{pept}} - \mathrm{H_{na}}]$	
	$c\,[\mathrm{C_{pept}} - \mathrm{O_{solv}}]$	

Table II: Sets of collective variables (CVs) used for the metadynamics simulations of the individual reaction steps according to the nomenclature introduced in Fig. 1 of the main text. The corresponding functional forms of the CVs are defined in Equations (3.3), (3.4), and (3.5) together with the distance cutoff parameters provided in Table I whereas the associated metadynamics sampling parameters are given in the text. The atomic symbols in square brackets belong to the molecule or functional group specified by the associated subscript: 'Gly' (glycine), 'COS' (carbonyl sulfide) 'COOH' (carboxyl group), 'COSH' (thiocarboxyl group), 'NCA' (glycine *N*–carboxyanhydride), 'pept' (peptide group), 'iso' (isocyanic group), 'na' (non–aliphatic), and 'solv' (solvent).

walkers are allowed to diffuse in the space of the CVs for a short time to ensure a better sampling of the FES.

Due to the finite size of the Gaussians added during metadynamics sampling, the filled potential can be locally rough, and thus the error in the free energy estimate depends on both the height and the width of the Gaussians. The height of the Gaussians will be discussed in the following section.

The dimensionless width parameter δs is set to 0.05, which is $\approx 1/4^{\mathrm{th}}$ of the width of the fluctuation of a CV type with the smallest amplitude oscillation in our simulations. The anisotropic scaling parameters $\{w_\alpha\}$, which have the units of the CVs, are determined in such a way that

$$w_\alpha = \frac{\max\left(|S_\alpha - \langle S_\alpha \rangle|\right)}{\delta s} \quad \forall \alpha \tag{3.6}$$

is satisfied, which is estimated during the preparation runs without adding any Gaussians. The coupling constants k_α allowed the CVs S_α and the associated auxiliary variables s_α to move close to each other. In conjunction with a judicious choice of the mass of the auxiliary variable μ_α, an adiabatic separation of $\{s_\alpha\}$ from the electronic degrees of freedom was maintained throughout all simulations. The values of k_α and μ_α depend on the type of CV and were chosen as 0.4 a.u. and 50 a.m.u., respectively, for distances and 2.0 a.u. and 50 a.m.u., respectively, for coordination numbers.

The metadynamics time step Δt^{MTD} is chosen adaptively during the dynamics in such a way that a Gaussian is placed at time t_i once the following condition is fulfilled

$$|s(t) - s(t_i)| = 3/2\, \delta s \ , \tag{3.7}$$

which is necessary to avoid so–called “hill-surfing” problems as discussed in Ref. 33. Note that steepness in the rough biasing potential may result in heating–up the system, because the auxiliary variables pull on the ionic system and thus accelerate the nuclei as they “roll down a hill”. Thus, it is necessary to keep the temperature of the auxiliary variables close to that of the physical system, i.e. the thermostatted nuclei. Due to the strong nonequilibrium nature of the dynamics of the auxiliary variables $\{s_\alpha\}$, simple velocity scaling is used to restrict the fluctuations of their fictitious kinetic energy corresponding to $\pm\,200\,\mathrm{K}$ with respect to the target temperature, while the nuclear degrees of freedom are controlled by massive Nosé–Hoover chain thermostats [22], which implies to couple one independent Nosé–

Hoover chain thermostat to each degree of freedom of each unconstrained atom in the system, to establish the canonical ensemble.

It is often the case that only some subregions of the space spanned by the CVs are of interest. In such cases, additional repulsive potentials (so-called 'wall potentials') are applied to some of the auxiliary variables in order to limit the exploration of the FES to the desired subregion, thus easily improving the sampling efficiency of the method. The wall potentials used in this study are compiled in Table II. For instance, two walls were applied in reaction **B**: the first one acts for $d\,[\mathrm{N_{Gly}} - \mathrm{C_{COS}}] \geq 4.2$ Å, thus the FES was sampled only for $d\,[\mathrm{N_{Gly}} - \mathrm{C_{COS}}] < 4.2$ Å, thereby avoiding that the reactants diffuse too far away from each other to form a reactive encounter complex. The second wall acts for $c\,[\mathrm{N_{Gly}} - \mathrm{H_{na}}] \geq 2.4$, which limits the values of $c\,[\mathrm{N_{Gly}} - \mathrm{H_{na}}]$ below 2.4 thereby keeping the amino group of glycine in its unprotonated state. However, the use of such repulsive walls can introduce an artificial deepening of the FES in the positions in which these are applied [33]. Consequently, these regions must be carefully avoided when computing free energy barriers.

D. Checking the Convergence and Error Bar Estimates

In previous works [12] it was determined that the best way to balance the efficiency and accuracy is to first fill the free energy well by using large Gaussian heights of $\approx 2{-}4\,k_{\mathrm{B}}T$ while keeping an appropriately narrow Gaussian width. Once a barrier crossing event is observed, the free energy estimate is refined subsequently by restarting the simulation sometime before this event occurred using comparatively smaller Gaussian heights of at most $1\,k_{\mathrm{B}}T$. The error of the free energy estimates obtained by this procedure has been shown to amount to about one $k_{\mathrm{B}}T$ (see the reference [77] in Ref. 12). Since smaller biasing potentials are used during the refinement, the system has more time to relax but the simulation time increases. The latter effect is not necessarily a disadvantage because it helps, together with any abnormal convergence behavior in the barrier height and mechanism during this procedure, to detect if any slow modes are missing in the set of CVs, and thus if the subspace as determined by their very definition can in fact accommodate the true reaction coordinate.

Even though this procedure has been proven accurate enough for the bulk solvent conditions (AMB and HPW), in the simulations under nanoconfinement (NCW system) we took additional measures to ensure a proper sampling of the reaction space and also checked

carefully that the reported free energy barriers were indeed converged. In the first place, we used a quite large number of walkers (between 8 and 32 depending on the reaction step) within the multiple walker scheme. In almost all reactions, a 'hierarchical' scheme has been used for the initialization of the walkers: the simulation begins by creating a small number of walkers (usually 4) from the previously equilibrated state and, as the simulation proceeds and the walkers diffuse across the CV space, more walkers are initialized by 'cloning' the existing ones and randomizing the velocities of the auxiliary variables, akin to what is done at the beginning of the simulation. The only exception is the pre-reactive equilibrium **A** in which we used 32 walkers from the beginning. We also note that the use of massive Nosé–Hoover thermostats [8, 22], in addition to providing a fast equilibration of the system, ensures a very fast (fictitious sampling) dynamics of all walkers in CV space. Even when starting from the same equilibrated configuration and randomizing only the velocities of the fictitious CVs, all the walkers are found to quickly decouple not only considering the modes that are explicitly driven by the CVs, but also considering the displacements of *all* atoms in the liquid phase as it is demonstrated by Fig. 2.

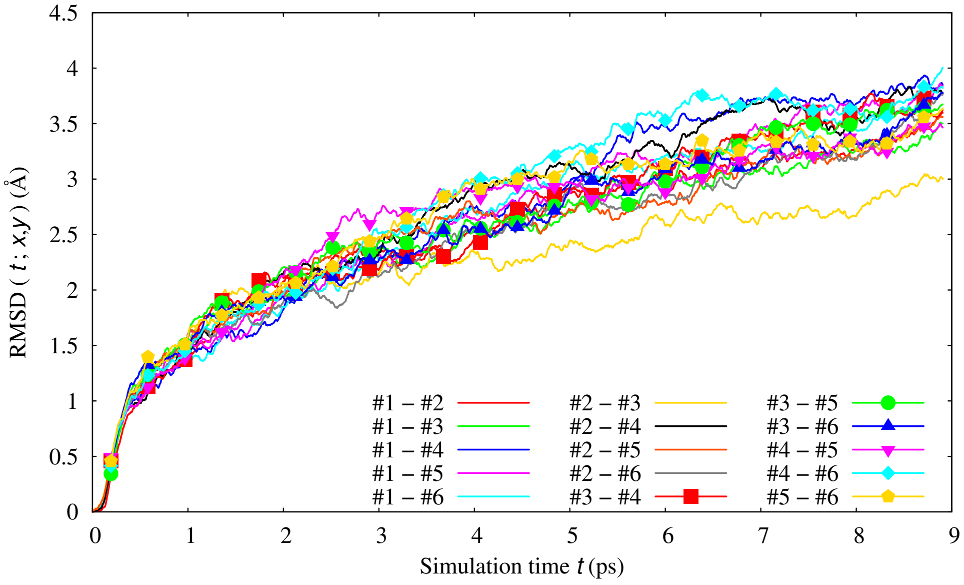

Figure 2: Relative root mean square deviation for all possible combinations of the first 6 walkers in reaction **A**, computed as $\mathrm{RMSD}\,(\,t\,;\,x,y\,) = \sqrt{\frac{1}{N}\sum_{i=1}^{N}|\mathbf{x}_i - \mathbf{y}_i|^2}$ where $\mathbf{x}_i$ and $\mathbf{y}_i$ are the cartesian coordinates of atom i in walkers $\mathbf{x}$ and $\mathbf{y}$ at the simulation time t, and the sum runs over all N atoms in the liquid phase. The remaining 26 walkers in reaction **A** show similar behavior.

Secondly, depending on the particular reaction step, we initialized metadynamics sampling using larger Gaussian heights ($\approx 2.5\, k_{\mathrm{B}}T$) before switching to smaller Gaussians ($< 1\, k_{\mathrm{B}}T$) *much before* the respective free energy well got filled up, while in other simulations small Gaussians were used during the whole metadynamics run which leads to similar results. Both, the number of walkers used in each simulation and the maximum (i.e. at the beginning of the simulation) and minimum (i.e. at the end of the simulation) values for the Gaussian heights are compiled in Table III.

Reaction	# of Walkers	Max./Min. Gaussian Height ($k_{\mathrm{B}}T_{500}$)	Simul. Time (ps)
**A**	32	0.16 / 0.08	274
**B**	2/4/8	2.5 / 0.63	47
**C**	4/16/32	0.63	218
**C'**	4/8/16/32	1.26 / 0.95	201
**D**	4/8/16	1.9 / 0.25	119
**E**	4/8/16	0.95 / 0.63	149
**F**	8/16/32	0.95	79
**G**	4/8/16/32	0.95 / 0.81	163

Table III: Number of walkers employed (which increases along the simulation according to our hierarchical approach, see text), maximum and minimum height of the Gaussians in $k_{\mathrm{B}}T_{500}$ units, and accumulated simulation time (being the accumulated sum of the simulation time of all walkers) for all reaction steps.

For irreversible reactions (**C**, **C'**, **E**, **F** and **G**), the simulation was terminated when the desired product was obtained in one or more walkers, and then only this walker is allowed to continue running until the free energy basin of the product state is well enough sampled on the FES in order to compute the minimum free energy path that links the reactant and product minima.

For reversible reactions (**A**, **B** and **D**), the protocol is slightly different. In reactions **A** and **D**, the simulations were prolonged so that several recrossings from/to reactants/products were observed, decreasing more and more the height of the Gaussians. As the simulation proceeds, the FES was reconstructed at certain intervals and the free energy barriers of the reaction were computed in each case to estimate reliably error bars for the particular

reactions in nanoconfined space.

For reaction **A**, we depict in Figure 3 (a) the evolution of all 32 walkers employed. It is clear that all the walkers have recrossed from the reactant to the product regions in the FES many times along the whole metadynamics run. In Fig. 3 (b), we plot the values of the free energies of the neutral form of glycine **2** and the transition state for the zwitterionic–neutral interconversion, both relative to the zwitterionic form **1**, computed along the simulation (note that the free energy of the partially ionized intermediate **1.1** is always very close to that of **1** and thus it is not shown here). Both ΔF values oscillate within a range of about 10 kJ/mol, which implies an error bar of ±5 kJ/mol ≈ ±1.2 $k_\mathrm{B}T_{500}$, which fully agrees with our previous estimates [12]. What is more important, the relative difference between them is reassuringly stable along the simulation and the free energy profile remains qualitatively the same even when *triplicating* the total accumulated simulation time (which is ≈ 93 ps for the first points in Fig. 3 (b) and ≈ 274 ps at the end of the run). The final reconstructed FES and the minimum free energy path are shown in Fig. 3 (c).

A similar analysis was carried out for reaction **D**. In Fig. 4 (a), many recrossings between the different states are observed for all employed walkers. The free energy differences for the different states relative to the isocyanate **4** do not change much upon increasing the accumulated simulation time from ≈ 48 ps (first points in Fig. 4 (b)) to ≈ 119 ps (end of the simulation), and their relative differences remain again stable even when constantly reducing the Gaussian height or increasing the number of walkers. This robust convergence behavior is thus very similar to what has been observed for reaction **A**. Even when comparing the most extreme oscillation in free energy values (between the point marked as (I), in which the simulation cannot yet be considered converged, and point (III)), the largest error bar amounts to ≈ ±8 kJ/mol ≈ ±1.9 $k_\mathrm{B}T_{500}$. A more realistic estimate obtained from comparing points (II) and (III), for which the free energy differences are better converged, yields an error estimate of ≈ ±4 kJ/mol ≈ ±1 $k_\mathrm{B}T_{500}$. Again, similarly to what has been found for **A**, what is more important is the fact that comparing the final free energy profile (IV) in Fig. 4 (c) to the profiles obtained at earlier stages of the simulation (II and III) demonstrates that the profile is *qualitatively consistent* in terms of the relative stability of intermediate and transition states as well as the mechanism of the reaction (i.e. stepwise as opposed to concerted). Thus, these key features are stable and do not change when increasing the simulation time or refining the profile by decreasing the Gaussian height.

For reaction **B**, we sampled one full recrossing (i.e. from reactant via product back to reactant). Given the large free energy differences between the states in this reaction and based on the observations of convergence for reactions **A** and **D** even with much less sampling than required for a meaningful error estimation, we can safely assume that further refinement would not qualitatively change the obtained free energy profile. The same holds

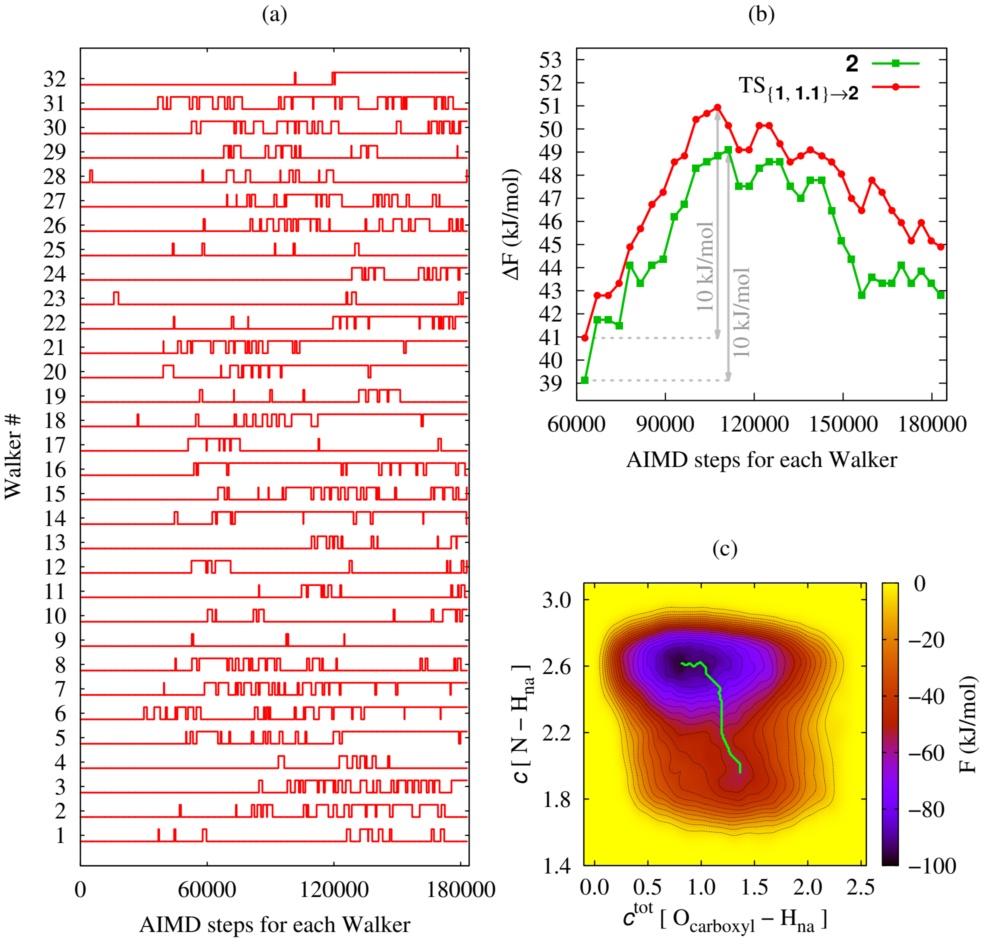

Figure 3: (a) Evolution of the walkers during the simulation of **A**. All walkers start from the zwitterionic state **1** and we represent their evolution as a step function in which 'step up' and 'step down' represent crossings from the zwitterionic to the neutral form and *vice versa*. (b) Free energy difference between zwitterion **1** and neutral species **2** of glycine (green squares) as well as the corresponding activation free energy (red circles) as a function of sampling steps; the same maximum error bar estimate of about ±5 kJ/mol as indicated is obtained for both relative free energies. (c) Reconstructed FES at the end of the simulation. The minimum free energy path is depicted in green.

true for the irreversible reactions **C**, **C’**, **E**, **F** and **G** for which the free energy profiles are also stable within the estimated error bar.

Finally, the free energy profiles of Fig. 2 in the main text are reproduced in Fig. 5 including the estimated error bars (but omitting the labels as well as the data points themselves).

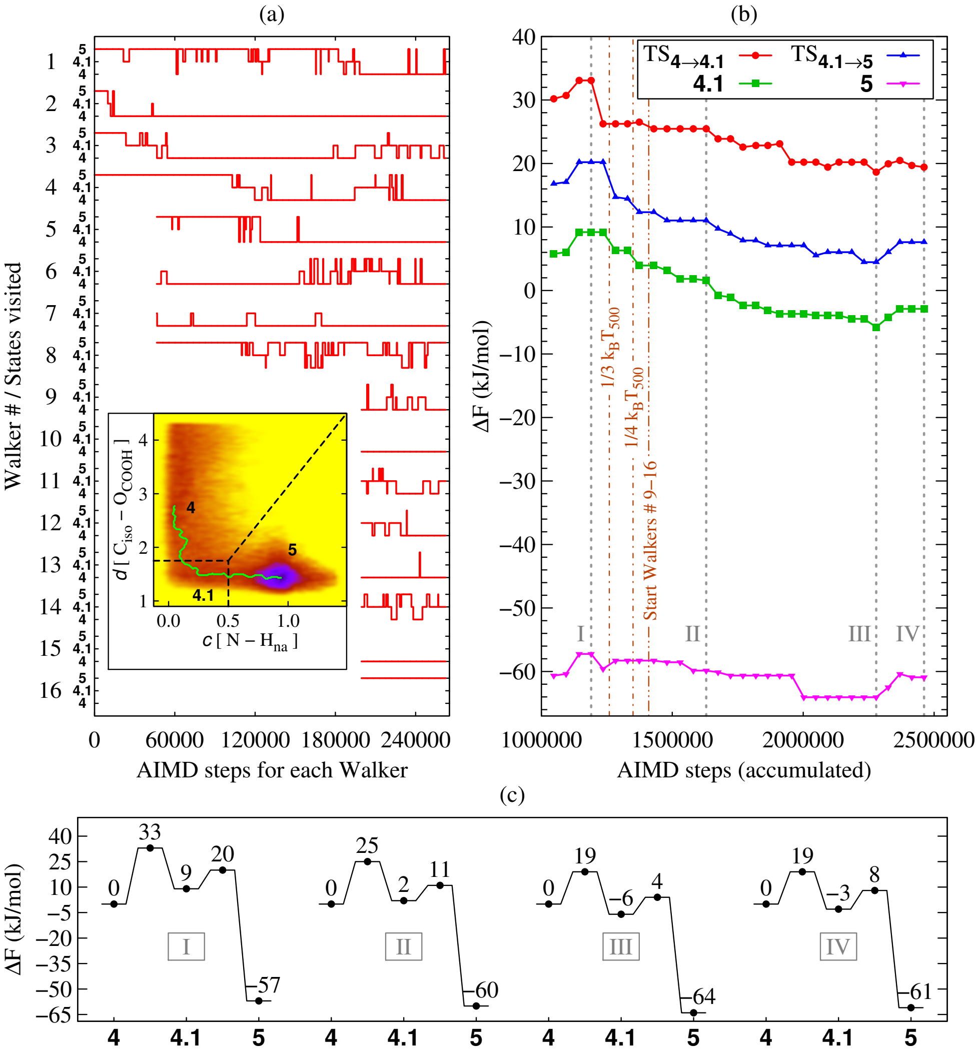

Figure 4: (a) Evolution of the walkers during the simulation for **D**. The simulation starts with walkers # 1-4 in the NCA state **5** and the rest of the walkers are initialized during the simulation by ‘cloning’ the previous ones, see text. The inset shows the reconstructed FES at the end of the simulation with the dashed lines indicating the regions belonging to each state. (b) Estimated free energy differences with respect to reactant **4** along the simulation indicating the changes in the employed Gaussian height (for the first five points it is 2/3 k_BT_{500}) or number of walkers. The sampled free energy profiles obtained at sampling points I–IV (marked by vertical dashed lines) are shown in (c) for clarity.

These profiles are discussed in the following with the focus on the distinct differences which distinguish the specific reaction step at NCW, HPW and AMB conditions in the spirit of the analyses, discussions, and conclusions as presented in the main text.

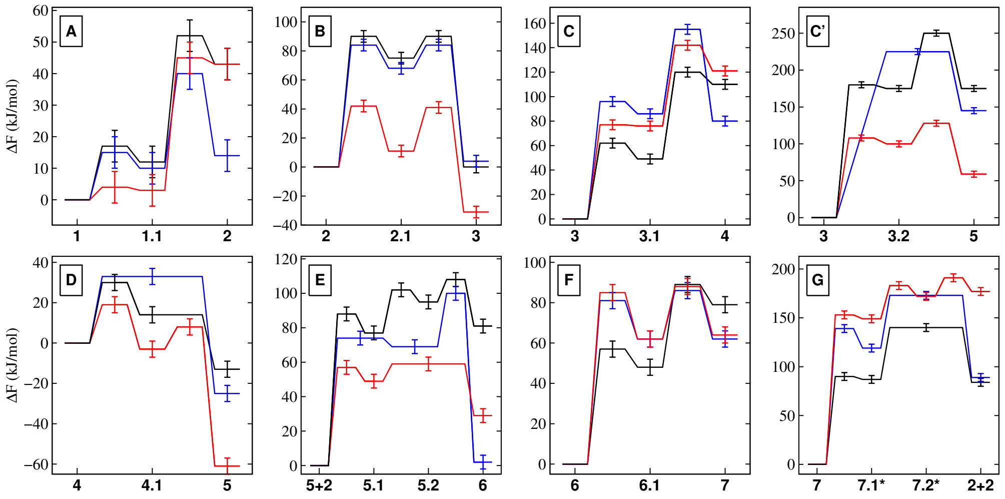

Figure 5: Free energy profiles of all reaction steps (reproduced from Fig. 2 in the main text except for labels and data points) including the estimated error bars for AMB (black), HPW (blue) and NCW (red) reaction conditions. Note the vastly different energy and thus error bar scales when comparing data in different panels to each other.

Reaction **A**:

The most important feature regarding this step of the reaction cycle is the free energy of the neutral form of glycine in water relative to the zwitterionic species for the different conditions. It is clearly seen that AMB and NCW are very close concerning that free energy difference, whereas both are qualitatively different from HPW. This supports our conclusion that nanoconfinement effects stabilize charge-separated species in water at given thermodynamic conditions.

Reaction **B**:

Considering the error bars, it is clear that the barrier for the forward reaction in NCW is about half of that in AMB/HPW, while the barrier for the back reaction is similar for all three conditions. Moreover, the charge-separated intermediate is more stable in NCW than in AMB/HPW.

Reactions **C** and **C'**:

Here, the error bars are almost negligible considering the large free energy differences and barriers involved in these steps.

Reaction **D**:

The most important features here are the concerted character in HPW *versus* the stepwise character in both AMB/NCW in addition to a clear stabilization of the intermediate **4.1** in NCW with respect to HPW. Considering the error bars, even in the worst-case scenario the reaction would remain stepwise in NCW, with the only difference that the intermediate region on the free energy surface might be a shallow minimum in this limit. Nevertheless, this species would be separated from the reactant **4** even in this most unfavorable scenario by a barrier that is similar to that observed in AMB, which is clearly different from the picture obtained in HPW.

Reaction **E**:

Here it is possible within the mutual error bars that the two transition states and the intermediate in NCW have the same free energy given that their error bars almost overlap according to the worst-case assumption. However, even if the free energy profile in NCW were essentially flat this would not change our analysis since the conclusion for this reaction is a lower barrier in NCW compared to AMB/HPW (which is indicative of the steric factors governing this particular reaction in NCW, see main text), which remains true even considering all error bars.

Reactions **F** and **G**:

Inclusion of the error bars do not alter the analysis for any of these reactions. In **F**, there is no remarkable change as the profiles in NCW and HPW are still equivalent and in **G** the main features of the reaction (i.e. higher barrier and stepwise character in NCW) are still clearly visible.

In summary, the analyses, discussions, and conclusions of the characteristic differences of nanoconfined versus bulk water as presented in the main text are supported for each individual reaction step when considering the mutual error bars.

IV. DIELECTRIC PROPERTIES OF NANOCONFINED WATER LAYERS

As stressed in the main text, the dielectric properties of interfacial water are known to be distinctly different from those of the bulk [6]. It is noted in passing that the local dielectric properties of such inhomogeneous liquid/solid systems [6, 7] have not been derived within the framework of continuum (implicit) solvation approximations, but that they are based on statistical mechanics of explicit solvent using a Kubo-like response approach as encoded in suitable correlation functions. Indeed, it is well know that continuum approaches, in particular convenient Born-type solvation models, typically fail to realistically describe the molecular solvation properties of hydrogen-bonding liquids such as water [34]. In addition, continuum solvation approaches split the total phenomenon into different contributions which requires approximate treatments of effects such as cavitation and electrostriction among several others [35], whereas solvation shell arrangements as well as solute-solvent interactions including hydrogen bonding are automatically included in fully explicit molecular approaches. Moreover, incorporating the effect of solvent on transition states of liquid-state chemical reactions is highly approximate without considering explicitly the intermolecular interactions between solute and solvent molecules, in particular if the solvent is actively involved in the chemical process [35]. It is in that sense that the chemical reactions studied herein at interfaces are certainly worst-case scenarios for applying any sort of implicit solvation model.

Upon computing the dielectric tensor based on very long molecular dynamics simulations using explicit water in contact with surfaces, the parallel component of this tensor is found to be considerably higher at the interfacial region, $\varepsilon_{\|}(z) \approx 150$, compared to sufficiently far away from the surface, $\varepsilon_{\|}(z) \approx 70$, for an hydrophobic model interface using the SPC/E water model [6]. Its (inverse) perpendicular component, $\varepsilon_{\perp}^{-1}(z)$, features a more complex behavior, but this turns out to be irrelevant as will be shown based on the subsequent analytic considerations. In the following we will determine to what extent the dielectric properties of interfacial water are dominated by the high value of the parallel dielectric response. The result applies even more to our nanoconfined water system given that it contains exclusively interfacial water for the layered mineral setup that is used in the present study.

In the following we stick to the same notation as used in Refs. 6, 7 where $\boldsymbol{m}(\boldsymbol{r})$ is the local polarization density comprising contributions from dipole, quadrupole and all high-order moments whereas $\boldsymbol{M} = \int_V \boldsymbol{m}(\boldsymbol{r})\, d\boldsymbol{r}$ is the resulting total polarization of the entire system.

At a planar interface between a solid and a liquid phase, the corresponding anisotropic boundary conditions impose that the dielectric tensor is diagonal with two unique components, $\varepsilon_{\|}(z)$ and $\varepsilon_{\perp}(z)$ being parallel and perpendicular to the interface, respectively, which are given here as a function of the distance z normal to the solid surface. Using these components, also $\boldsymbol{m}(\boldsymbol{r})$ and thus $\boldsymbol{M}$ can be expressed in terms of their parallel and perpendicular components, i.e. $m_{\|}(z)$, $m_{\perp}(z)$, $M_{\|}$ and $M_{\perp}$. We start our analysis with Eqs. (26) and (28) provided in Ref. 7, i.e.

$$\varepsilon_{\|}(z) \approx 1 + \varepsilon_0^{-1}\beta[\langle m_{\|}(z)M_{\|}\rangle - \langle m_{\|}(z)\rangle\langle M_{\|}\rangle] \tag{4.1}$$

$$\varepsilon_{\perp}^{-1}(z) \approx 1 - \varepsilon_0^{-1}\beta[\langle m_{\perp}(z)M_{\perp}\rangle - \langle m_{\perp}(z)\rangle\langle M_{\perp}\rangle] \ , \tag{4.2}$$

which provide the two distance–resolved components of the dielectric tensor in terms of the components of the local and total polarizations for planar inhomogeneous systems; here ε_0 is the vacuum dielectric constant and $\beta = 1/k_{\mathrm{B}}T$. Since $\langle \boldsymbol{M}\rangle = \boldsymbol{0}$, this leaves us with

$$\varepsilon_{\|}(z) \approx 1 + \varepsilon_0^{-1}\beta\langle m_{\|}(z)M_{\|}\rangle \tag{4.3}$$

$$\varepsilon_{\perp}^{-1}(z) \approx 1 - \varepsilon_0^{-1}\beta\langle m_{\perp}(z)M_{\perp}\rangle \tag{4.4}$$

from which

$$\langle m_{\|}(z)M_{\|}\rangle \approx \frac{\varepsilon_{\|}(z) - 1}{\varepsilon_0^{-1}\beta} \tag{4.5}$$

$$\langle m_{\perp}(z)M_{\perp}\rangle \approx \frac{1 - \varepsilon_{\perp}^{-1}(z)}{\varepsilon_0^{-1}\beta} \tag{4.6}$$

follows immediately. Now we can combine both relations to express the ensemble average of the polarization fluctuations,

$$\langle \boldsymbol{m}(z)\boldsymbol{M}\rangle = 2\langle m_{\|}(z)M_{\|}\rangle + \langle m_{\perp}(z)M_{\perp}\rangle \approx \frac{2\varepsilon_{\|}(z) - \varepsilon_{\perp}^{-1}(z) - 1}{\varepsilon_0^{-1}\beta} \ , \tag{4.7}$$

depending on the distance–resolved values of the parallel and the *inverse* of the perpendicular component of the dielectric tensor within these boundary conditions; note that this equation holds locally within the entire x/y–plane at height z above the surface. This distance–dependent expression for $\langle \boldsymbol{m}(z)\boldsymbol{M}\rangle$ is the planar anisotropic analog of the usual homogeneous isotropic formula for bulk liquids [36, 37], written in SI units as

$$\langle \boldsymbol{m}\cdot\boldsymbol{M}\rangle = 3\,\frac{\varepsilon_{\mathrm{bulk}} - 1}{\varepsilon_0^{-1}\beta} \ , \tag{4.8}$$

that directly relates the static bulk dielectric constant to the polarization fluctuations in the bulk in terms of the second moment of the total dipole moment distribution $\langle \boldsymbol{M}^2 \rangle$ (noting that $\boldsymbol{m} = \boldsymbol{M}/V$ in the homogeneous isotropic case). As such, the anisotropic polarization fluctuations $\langle \boldsymbol{m}(z)\boldsymbol{M} \rangle$ are related to the ability of water to host polarized or charge-separated species as a function of the distance z of these species normal to the solid surface.

As shown in Refs. 6 and 7, $\varepsilon_{\|}(z)$ reaches an enormously high value of ≈ 150 at the interface with an hydrophobic solid surface or ≈ 120 at an hydrophilic one (whereas it is ≈ 70 far from the surface), while $\varepsilon_{\perp}^{-1}(z)$ is always in the range of about -1 to $+1$. Therefore, the contribution of $\varepsilon_{\perp}^{-1}(z)$ can be safely neglected close to the interface compared to the parallel component, $\varepsilon_{\|}(z)$. This implies that the polarization fluctuations close to the interface are completely dominated by the parallel component,

$$\langle \boldsymbol{m}(z)\boldsymbol{M} \rangle \sim \frac{\varepsilon_{\|}(z)}{\varepsilon_0^{-1}\beta} \ , \tag{4.9}$$

and are thus much higher there than further away from the surface, where $\varepsilon_{\|}(z)$ quickly approaches ≈ 70 according to the SPC/E water model used in Refs. 6, 7; note that the enhancement of $\varepsilon_{\|}(z)$ in interfacial water has been described in other studies as well [38, 39]. On the other hand, in contrast to the clear enhancement of $\varepsilon_{\|}(z)$ observed for water at hard interfaces, only a very modest enhancement has been found in water confined by soft interfaces [40].

Thus, if something like a "local dielectric constant of interfacial water" could be defined (in view of the stunning similarity of Equation (4.9), which is valid only close to the confining surface, and Equation (4.8) being approximately valid in bulk water in the form $\langle \boldsymbol{m} \cdot \boldsymbol{M} \rangle \sim \varepsilon_{\text{bulk}}/\varepsilon_0^{-1}\beta)$, this dielectric constant would be completely dominated by the overridingly large parallel component of the dielectric tensor close to the surface because of the great enhancement of the average polarization fluctuations $\langle \boldsymbol{m}(z)\boldsymbol{M} \rangle$ of interfacial water.

Finally, when it comes to the energetic impact of such enhanced polarization fluctuations on the solvation of charged species in liquids, we can refer to a recent detailed analysis carried out for simple ions in bulk water [41]. In particular, the following linear response expression has been derived [41] for the energy change due to the field that is created by spherical solute species of point charge q surrounded by a spherical water drop of radius R,

$$\Delta U = - \ \frac{\beta}{2\,\varepsilon_0^2} \int_0^R q^2 \left[\langle m_{\text{rad}}(r) M_{\text{rad}} \rangle - \langle m_{\text{rad}}(r) \rangle \langle M_{\text{rad}} \rangle \right] dr \ , \tag{4.10}$$

based on the radial components, M_{rad} and $m_{\text{rad}}(r)$, of the usual total polarization and local polarization density; as before $\langle \ldots \rangle$ denotes the statistical-mechanical ensemble averages in the *absence* of the field that is created by the ionic charge and again $\langle \boldsymbol{M} \rangle = \mathbf{0}$. The electrostatic stabilization is well-known to dominate the solvation free energy of charged species, such as ions, in liquid water [42]. The above expression, therefore, makes transparent the direct link between the magnitude of the polarization fluctuations of water and the electrostatic contribution to the solvation free energy of charged species; note that we are not even attempting to use an Equation such as (4.10) to *quantify* the solvation free energies of the multi-molecular reactive complexes along their complex reaction pathways including transition states and short-lived intermediates in interfacial water, but only to *qualitatively* establish the favorable impact of large polarization fluctuations on the stabilization of charged species in solution, without addressing other details such as the influence of the confining medium which are out of the scope of this study, even though these may be significant and should be investigated in further works. In this context, we stress that also this relation does not rely on any dielectric continuum approximation since the statistical-mechanical thermal averages are obtained from fully atomistic molecular dynamics simulations.

V. DETAILED RESULTS FOR ALL REACTIONS OF THE PEPTIDE CYCLE

Reaction A: Glycine Zwitterionic Equilibrium.

The overall free energy barrier for the zwitterionic to neutral interconversion of glycine is *slightly higher* in NCW than in HPW. Interestingly, the relative stability of the neutral species is similar to AMB such that NCW is found to *stabilize* the charged zwitterionic state akin to AMB (and unlike to HPW) – despite water being at high pressure and temperature conditions. Deprotonation/protonation of the amino and carboxyl groups in NCW is observed via either intramolecular concerted proton transfer or Grotthuss migration through the solvent. Most of the time the glycine can be seen nicely integrated in one of the water layers (Fig. 6), adopting a planar conformation parallel to the surface. This very conformation clearly favors concerted proton transfer from the $–NH_3^+$ (resp. –COOH) group to the $–COO^-$ (resp. $–NH_2$), either by direct proton transfer or mediated by a single solvent molecule in a one–step Grotthuss–like mechanism.

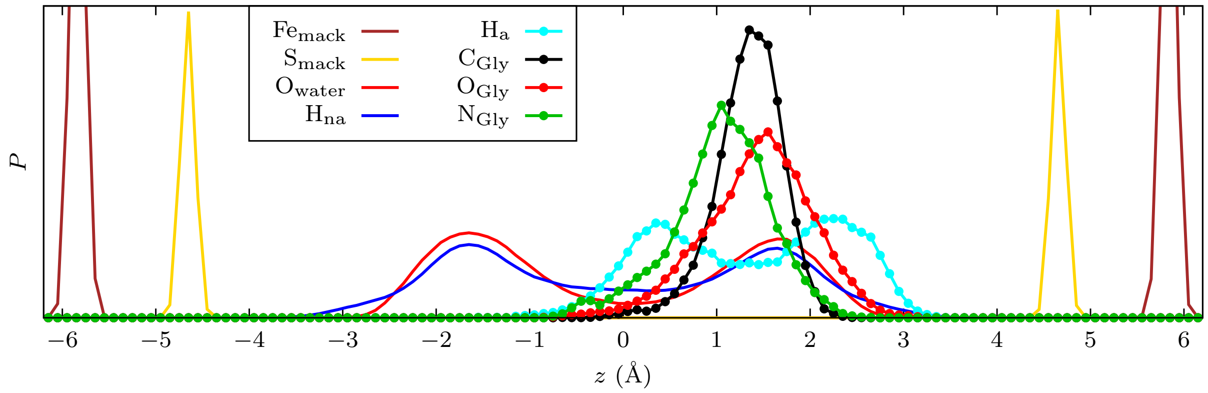

Figure 6: Normalized number density profiles for reaction **A** as a function of the distance perpendicular to the mineral surface ($z = 0$ indicates the midplane of the water bilayer system). The FeS mackinawite atoms are denoted by Fe_{mack} and S_{mack}; O_{wat} indicates all O atoms in the water phase and H_{na} are all non-aliphatic H atoms in the system (i.e. all H atoms in the system except those in the glycine $–CH_2–$ group which are denoted as H_a); the subscript 'Gly' identifies all the heavy atoms of glycine. This plot shows that the glycine molecule is nicely localized within one of the water layers and that the overall water bilayer structure of the nanoconfined lamella is preserved.

Reaction B: Glycine and COS Addition.

Let us start by analyzing this reaction step at AMB vs. HPW thermodynamic conditions with a particular focus on *all* charge/protonation states involved (Fig. 7) in view of the unexpectedly similar free energy profiles in ambient and hot-pressurized water (Fig. 2B in the main text). While the reaction mechanism as such is essentially the same in both cases (i.e. attack of the amino group of **2** at the C atom of COS leading to **2.1** followed by deprotonation of the NH_2^+ group to yield **3**), there is an interesting difference regarding the protonation state of glycine's carboxyl group. Importantly, this functional group is not involved in the specific chemical reaction **B** itself, but rather serves as a non-reactive 'spectator' group. The carboxyl group is found to stay in its neutral (i.e. –COOH) form for the whole reaction in case of HPW conditions, whereas in AMB it is in equilibrium between the protonated and deprotonated forms only in the reactant state (as is well known from weak acids such as amino acids in water at ambient conditions) but it is found to be deprotonated in both the intermediate **2.1** and product **3** states; such protonation equilibria are symbolized using $\{\ldots\}$ in this and all subsequent such reaction schemes. Consider now specifically **2.1** with the help of Fig. 7: this species carries a full charge in AMB, which is greatly stabilized by water given its dielectric properties at ambient conditions [43], whereas the carboxylate group remaining protonated at HPW conditions leaves us with an overall neutral species which favors solvation therein (compared to potentially solvating the same overall charged species **2.1** from AMB). Yet, the nascent amide group, being the reactive group in **B**, must stay protonated after addition of COS which yields the intermediate state **2.1**, independently from the thermodynamic and thus solvation conditions. Subsequently, **2.1** is stabilized, again independently on the thermodynamic conditions, by detaching the excess proton on the amide group thus leading to product **3**. However, given the different initial state **2.1**, species **3** is in equilibrium between doubly and singly negatively charged states in ambient water, while it is either neutral or carries at most one negative charge in HPW. Hence, the total charge state of the involved species is *clearly different* when comparing AMB to HPW, despite the fact that the two free energy profiles are *almost identical* at these distinctly different thermodynamic conditions. Since the former favors the formation of charged and charge-separated states, the latter favors neutral states in complete accordance to what it is known on the preferred solvation of charged vs. neutral

solutes based on the vastly different dielectric properties of bulk water at AMB vs. HPW conditions [43].

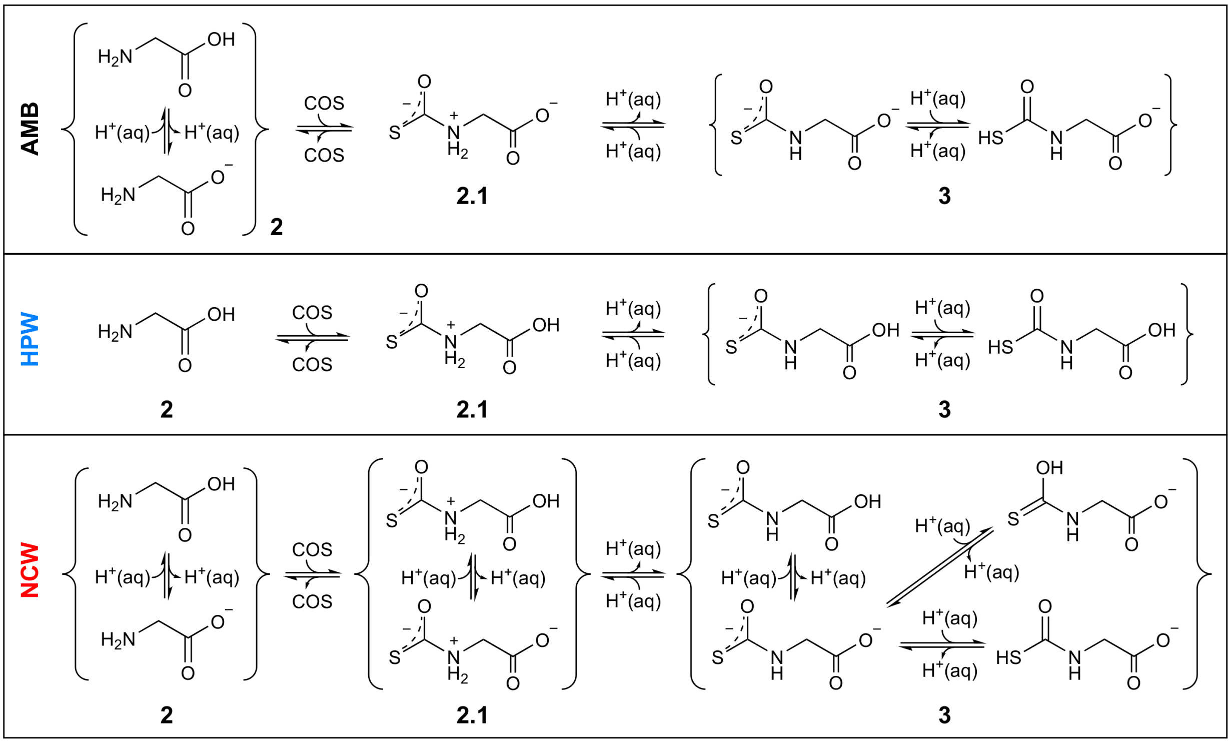

Figure 7: Detailed mechanism for reaction **B** at the three studied conditions.

In conclusion, being non-reactive in reaction **B**, the carboxyl group's protonation state is able to freely adjust to additionally favor solvation of the entire molecule depending on the conditions and thus acts as a 'buffer', whereas the protonation state of the reactive amide group is dictated by the reaction mechanism itself. This subtle effect results into surprisingly similar free energy profiles of reaction step **B** when taking place at AMB and HPW thermodynamic conditions, since the protonation state of the 'spectator' carboxyl group is not part of the reaction coordinate reflected in the free energy profiles but it is clearly affected by the changing solvation conditions.

Addressing next the NCW conditions, nanoconfinement induces no changes in the mechanism (Fig. 3 in the main text) also in this case, but the free energetics is strongly affected (Fig. 2B in the main text): the activation free energy for the key COS addition reaction leading to **2.1** is reduced by a factor of two to about 42 kJ/mol, while the charged intermediate **2.1** is further stabilized down to roughly 11 kcal/mol relative to the reactant state **2**, whereas the barrier for the reverse reaction back to **2** is only slightly lower than at AMB

and HPW conditions. During the whole simulation, both the COS and glycine are observed to remain integrated in one of the water layers, as depicted in Fig. 3 in the main text and Fig. 8 below. The big difference in NCW between the free energy barriers for the direct and reverse reactions is easily explained in terms of steric factors: while nanoconfinement favors the addition reaction compared to the bulk by restricting the translational freedom of the reactants and thus favoring reactive encounters, the elimination reaction is not affected by the dimensionality reduction intrinsic to nanoconfinement and its free energy barrier is thus very similar to that in the bulk regime.

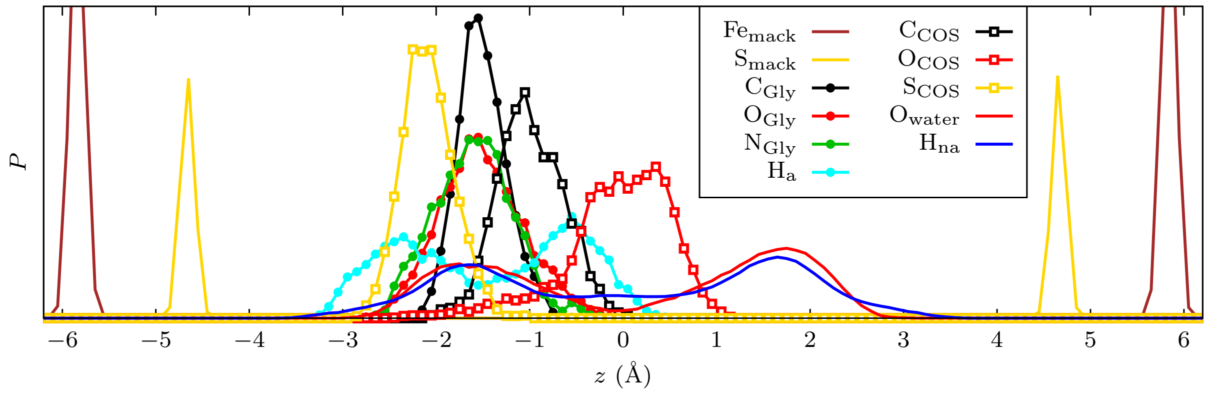

Figure 8: Normalized number density profiles for reaction **B** as a function of the distance perpendicular to the mineral surface ($z = 0$ indicates the midplane of the water bilayer system). The FeS mackinawite atoms are denoted by Fe_{mack} and S_{mack}; O_{wat} indicates all O atoms in the water phase and H_{na} are all non-aliphatic H atoms in the system (i.e. all H atoms in the system except those in the glycine $-CH_2-$ group which are denoted as H_a); the subscripts 'Gly' and 'COS' identify all the heavy atoms of glycine and COS. Akin to what was shown for reaction **A**, here both glycine and COS are localized within one of the water layers while at the same time the overall water bilayer structure of the nanoconfined lamella is preserved.

Coming now to the behavior of the spectator group in the NCW case, it is seen to be in equilibrium between its neutral (–COOH) and anionic ($-COO^-$) forms for *all* the involved species. It is noted in passing that the COS moiety in the product **3** is observed in all protonation states possible, being its anionic or neutral form after protonation at its sulfur or oxygen sites.

In conclusion, the resulting gross picture resulting from nanoconfinement effects is the

general trend that all functional groups are preferentially charged in NCW (if allowed within the constraints imposed by the specific reaction mechanism) much like at AMB conditions, but at variance with HPW where overall neutral species are preferred whenever this is possible. On the other hand, the influence of specific steric factors depending on the distinct chemical reaction is clearly revealed as well in the corresponding three free energy profiles and the resulting free energy differences as reported in Fig. 2B in the main text.

Reaction C: SH^- Elimination from *N*–Thiocarboxyl Glycine.

The stepwise elimination of SH^- from **3** is favored in NCW over HPW. Not only the intermediate **3.1** presents a lower free energy in the nanoconfined regime than in HPW, but also its formation requires to surmount a lower free energy barrier. Moreover, in addition to the stepwise mechanism we have observed a *concerted* reaction channel in NCW from **3** to **4** in which the *N*–deprotonation and SH^- elimination steps occur simultaneously, even though the free energy barrier for this process is ∼160 kJ/mol. Thus, stepwise elimination in NCW is clearly preferred over the concerted mechanism.

Regarding the protonation states of the different species, the carboxyl group stays in its neutral form for the whole reaction in HPW, while at both AMB and NCW conditions it is in equilibrium between the neutral and anionic form for all the states. The COS moiety is seen to adopt all possible protonation states in all AMB/HPW/NCW conditions, thus being in all cases in equilibrium involving its $–COS^-$, –COSH, and –CSOH forms.

Reaction C': Direct Cyclization of *N*–Thiocarboxyl Glycine.

The direct cyclization pathway of *N*–thiocarboxyl glycine **3** with SH^- elimination to form the *N*–carboxyanhydride **5** in NCW is a stepwise reaction via reaction intermediate **3.2** (Fig. 4 in the main text). This was also the case in AMB, while in HPW the reaction is concerted and **3.2** exists only as a transition state. The nanoconfinement also induces a dramatic decrease of the free energy barrier, from 225 kJ/mol in HPW to 128 kJ/mol in NCW. It is interesting to realize that in this reaction, where the charge state of the intermediate **3.2** is exactly the same in AMB, HPW and NCW (because the carboxyl group of **3** is chemically involved in the cyclization of **3** to NCA **5** and therefore no longer a

spectator that is free to adapt its protonation state depending on the conditions as it does in reaction **C**), the qualitative character of the reaction is the same in AMB and NCW but distinctly different to that in HPW.

Reaction D: NCA Formation by Isocyanate Cyclization.

Here again, nanoconfinement causes a change in the mechanism since the reversible cyclization of the isocyanate **4** to the NCA **5** in NCW proceeds in a stepwise manner through the intermediate **4.1**, which is only a transition state in HPW. This stepwise mechanism is similar to the one observed in AMB, where **4.1** is also an intermediate but less stable than in NCW. Overall, the nanoconfinement increases the shift in equilibrium towards the formation of the NCA **5**, which is the activated amino acid ready for peptidization, since the free energy barrier for the direct reaction is lowered whereas the one for the inverse reaction is increased at these conditions (Fig. 2D in the main text). As it is shown in Fig. 9, all species involved in the reaction step, i.e. **4**, **4.1** and **5**, are rather mobile within the water bilayer,

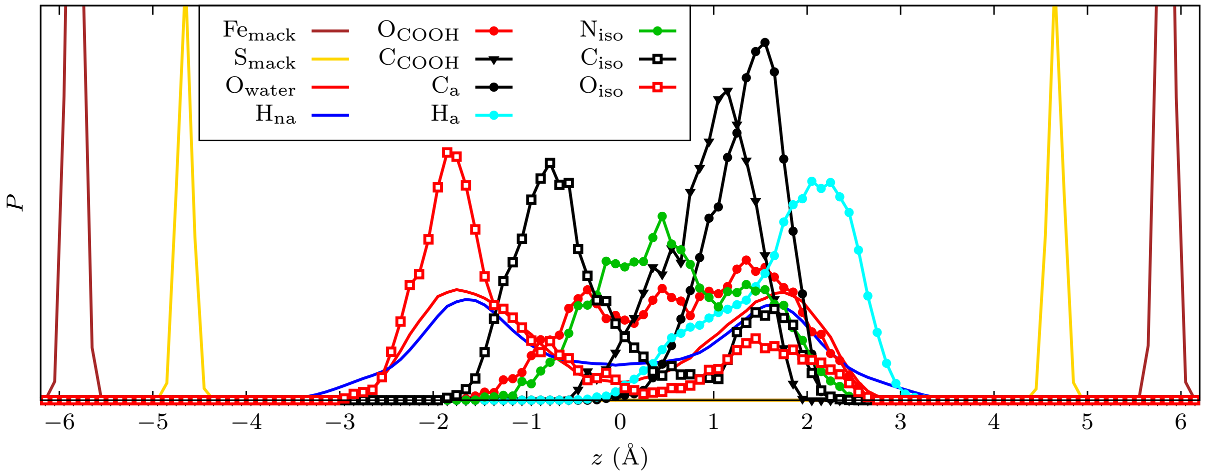

Figure 9: Normalized number density profiles for reaction **D** as a function of the distance perpendicular to the mineral surface ($z = 0$ indicates the midplane of the water bilayer system). The FeS mackinawite atoms are denoted by Fe_{mack} and S_{mack}; O_{wat} indicates all O atoms in the water phase and H_{na} are all non-aliphatic H atoms in the system (i.e. all H atoms in the system except those in the $-CH_2-$ group which are denoted as H_a; the aliphatic carbon is labeled as C_a); the subscripts 'iso' and 'COOH' identify the isocyanate (–NCO) and carboxyl groups.

can be either integrated in one one of the water layers (parallel to the mineral surfaces), or can adopt an oblique orientation across the whole nanoconfined space. In either case, the underlying water bilayer structure is clearly maintained in the density profiles of O_{wat} and H_{na} according to the results depicted in Fig. 9.

Like in reaction **C'**, it is seen also in **D** that when the intermediates have exactly the same charge state in the three studied conditions (because the carboxyl group again is unavailable for becoming engaged in protonation equilibria in order to favorably adjust the overall charge state of reacting species to the specific reaction conditions), the qualitative character of the reaction is the same in AMB and NCW and qualitatively different from HPW.

Reaction E: Peptidization.

This reaction proceeds by nucleophilic attack of the amino group of glycine on the C5 atom of the activated amino acid in form of its NCA, followed by ring opening and deprotonation of the N atom of the peptide bond (Fig. 10); it is noted in passing that the *cis* form of the peptide is obtained under all three reaction conditions. The relative stability of the involved species turns out to be very different depending on the reaction conditions, and so is the protonation state of the glycine carboxyl group. In NCW, glycine's carboxyl group in the reactant state **5** is in equilibrium in terms of its neutral and anionic forms, whereas it is exclusively deprotonated ($-COO^-$) and thus in its charged state for the chemical reaction that leads to **5.1**, as indicated by the arrow in the respective leftmost subpanel of Fig. 10, and also subsequently from **5.1** to species **5.2**, where **5.1** is a well-defined intermediate state. Once the ring in the NCA fragment is open, the N atom of the peptide bond quickly deprotonates and **5.2** is only transiently observed before formation of the product **6**. It is noted in passing that this last step is an intramolecular proton transfer, where the terminal carboxyl group that was in its anionic carboxylate form captures the excess proton of the N atom of the peptide bond (as indicated by the arrow that denotes the migration of the proton in the $[\ldots]^\ddagger$ scheme), which is favored by the compact conformation of the dipeptide that is enforced by the nanoconfinement. In turn, the resultant protonated and overall neutral form of the diglycine derivative **6** is seen to also deprotonate at its carboxyl terminus, in accordance with previous observations at NCW conditions.

This is the opposite scenario of what happens in HPW. Here, the glycine carboxyl group

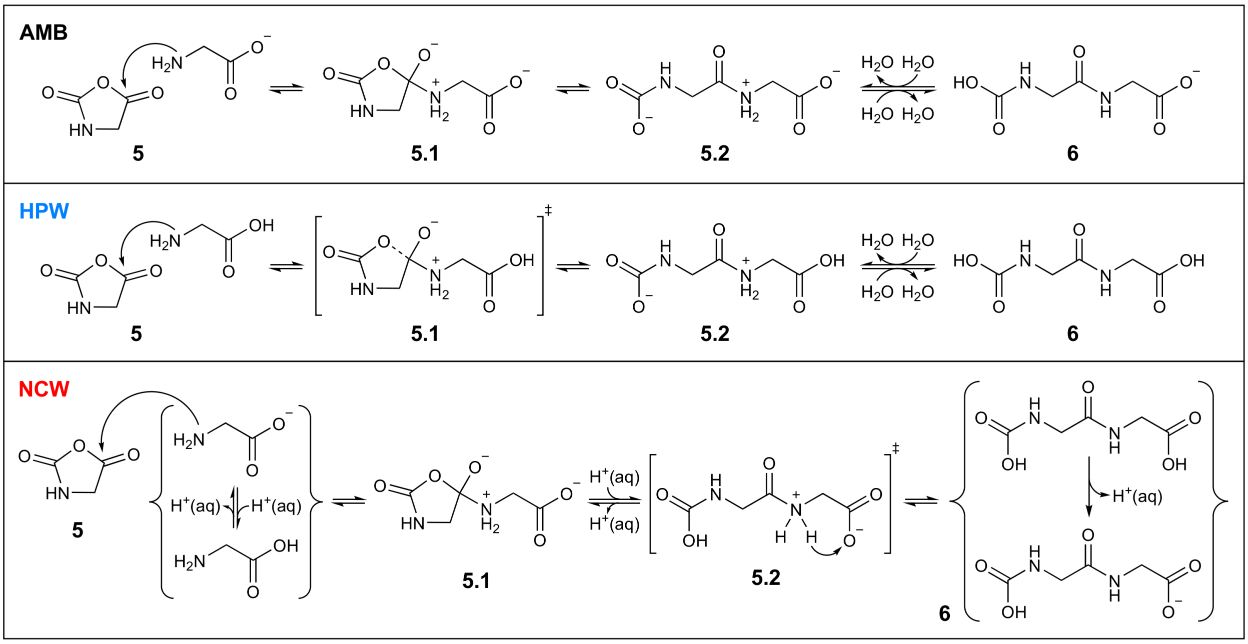

Figure 10: Detailed mechanism for reaction **E** at the three studied conditions where the arrows within the formulae symbolize the displacements of nuclei.

remains neutral along the whole multistep process **E**. The attack of glycine on NCA **5** triggers a fast ring opening where **5.1** is now the transition state, whereas **5.2** is a very stable intermediate which needs to surmount a considerable free energy barrier for deprotonation to yield the product **6**. Note, in addition, that in species **5.2** the carboxyl group of the carbamic acid (located at the 'left' terminus of the molecule in Fig. 10) remains anionic in HPW, thus keeping the molecule globally neutral in view of the protonated amide bond, until the neutral product **6** is formed.

In AMB, on the other hand, both **5.1** and **5.2** are stable intermediates, and glycine's carboxyl group remains in its deprotonated state $-COO^-$ for the whole reaction akin to what was observed at NCW conditions.

To wrap up, similarly to what happened in reaction **B**, the overall free energy barrier for this reaction is surprisingly similar in AMB and HPW, but the charge state of the involved species is remarkably different, again confirming the trend of extreme conditions to disfavor charged states compared to AMB. Nanoconfinement is found to strongly decrease the free energy barrier mostly because of steric factors favoring the attack of glycine on the NCA **5** and the subsequent purely intramolecular deprotonation of the central $-NH_2^+-$ amide group together with favoring the charged state of glycine's carboxyl group along the entire reaction

process – being again similar to the scenario in AMB and very distinct from what is observed at HPW conditions.

Reaction F: CO_2 Elimination.

Elimination of CO_2 to finally yield the dipeptide **7** proceeds according to a similar mechanism under all conditions: proton transfer from the O atoms of carbamate **6** to its N atom via Grotthuss diffusion, followed by the release of CO_2 from the intermediate **6.1** (Fig. 11). Again, interesting conclusions arise when analyzing the protonation state of the terminal carboxyl group stemming from the glycine in the previous reaction **E** (depicted at the right side of the species in Fig. 11), being now the easily de/protonable spectator group in **F**. At HPW conditions it stays in its neutral form for the whole reaction, while at AMB conditions it is initially at equilibrium between the anionic and the neutral form. However, at AMB the individual reaction steps from reactant **6** to intermediate **6.1** to product **7** occur while staying in the neutral form of the carboxyl group. Hence, in this case we can compare the impact of the changing conditions when the charge state of all the species at both AMB and HPW is exactly the same, unlike to what is observed in reactions **B** or **E**. In **F**, the

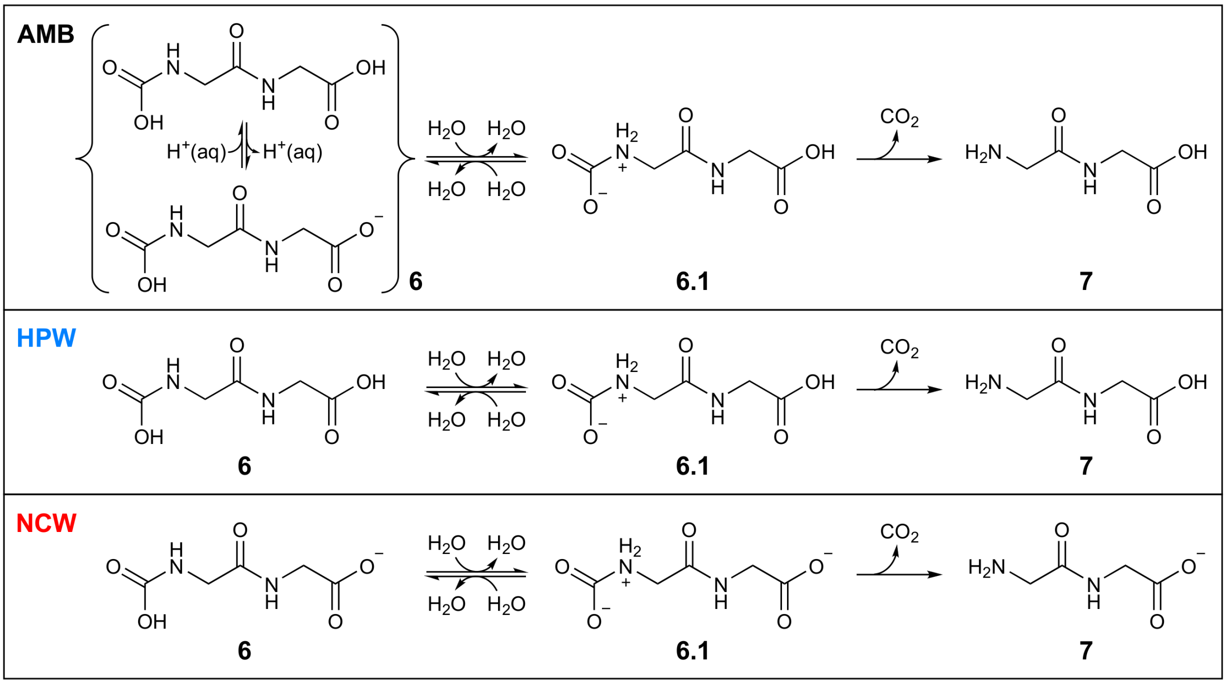

Figure 11: Detailed mechanism for reaction **F** at the three studied conditions.

free energy profiles (Fig. 2F of the main text) reveal that the formation of the intermediate **6.1** is quite disfavored at HPW compared to AMB conditions, as it is expected from the different dielectric properties of bulk water at both situations.

On the other hand, in NCW, this spectator carboxyl group remains anionic along the whole reaction sequence, which is completely the opposite picture of what happens at HPW conditions. The most revealing fact in this case is the realization that the free energy profile in NCW is almost identical to that in HPW, but throughout the whole reaction the different conditions impose a different overall charge state of the involved species due to the adjusted protonation state of the carboxyl/carboxylate spectator group.

In conclusion, these findings confirm the trend already observed in several other reactions. Upon changing the conditions from AMB to HPW (or from HPW to NCW), either the free energy profiles dramatically change as expected, which is observed in those cases where the overall charge state of the involved species remains the same at the different conditions (e.g. AMB vs. HPW in the present reaction **F** and also AMB vs. HPW *vs.* NCW in reactions **A**, **C'** and **D**), or else the free energy profiles remain quite similar but the charge state of the involved species is distinctly different as a result of suitable de/protonations of the spectator group in response to the different solvation conditions (e.g. HPW vs. NCW in the present reaction **F** or AMB vs. HPW in reaction **B**).

Reaction G: Peptide Hydrolysis.

This important back-reaction follows radically different mechanisms depending on the solvation conditions (Fig. 12). In NCW the first step is a direct attack of a water molecule to the peptide bond forming the highly polar intermediate **7.1"**. The excess proton at the bound water molecule is not immediately released but rather stays attached. Alternatively, it is shared with one of the solvent waters or with an O atom of the terminal carboxyl group (which in NCW essentially remains in its anionic form apart from some transient protonations as sketched in terms of equilibria in Fig. 12), in both cases rattling back and forth between the two oxygens akin to the shared proton in a Zundel complex (Fig. 5 in the main text). In the second step, the deprotonation of the attached water occurs as well as the protonation of the peptide's N atom thus forming the species **7.2"**. We have observed two distinct reaction channels for this step: in the first one, the reaction proceeds with

assistance by one of the solvation waters, which donates a proton to the peptide's N atom and captures the excess proton from the attached water molecule, being a Grotthuss–type proton migration of only one step (Fig. 5 in the main text). In the second pathway, the peptide's N atom receives a proton from a solvation water, which is followed by Grotthuss diffusion (via another solvent water) of the excess proton of the bound water to the terminal carboxylate group of the peptide. Once **7.2"** is formed, the rupture of the peptide bond itself presents a low barrier of only $\sim 5\ k_{\mathrm{B}}T_{500}$.

In stark contrast to this NCW scenario, the first step at HPW conditions is the attack of a water molecule on the peptide bond C atom with a concerted proton donation of this same H_2O to the terminal amino group of the dipeptide, thus leading from **7** to **7.1'** while avoiding a zwitterionic transition state $[\ldots]^{\ddagger}$ as sketched in Fig. 12. Subsequently, the now protonated amino group in **7.1'** donates back a proton to the N atom of the peptide bond in an intramolecular proton transfer event, thus breaking the dipeptide apart into two glycine molecules, **2** + **2**. It is important to note that both proton transfers observed in this case

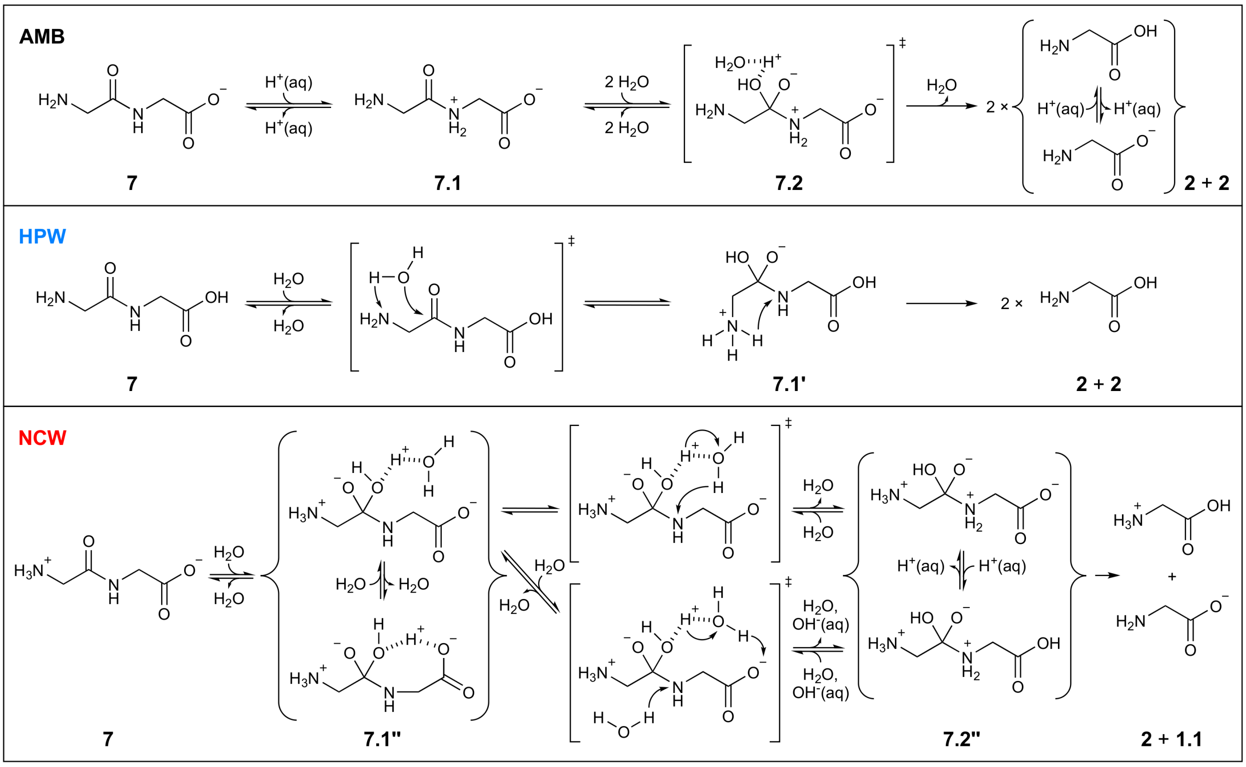

Figure 12: Detailed mechanism for reaction **G** at the three studied conditions where the arrows within the formulae symbolize the displacements of nuclei.

are intramolecular processes, i.e. not mediated by the solvent. In addition, the terminal carboxyl group of diglycine, being the spectator in **G**, remains in its neutral form during the whole reaction.

Finally, in AMB the first step is the protonation of the peptide's N atom, yielding the clearly zwitterionic intermediate **7.1** that accordingly carries a pronounced dipole moment due to the negatively charged carboxylate terminus in the presence of a protonated amide group (Fig. 12). Next, attack of a water molecule leads to the transition state **7.2**, which again is a charge-separated and thus zwitterionic species, where the attacking H_2O donates a proton to one of the surrounding waters with the subsequent breaking of the peptide bond again yielding two glycine molecules **2**. In stark contrast to HPW but similar to NCW, the terminal carboxyl group, being the spectator in **G**, remains in its anionic form along the full reaction.

Regarding the free energies, this back-reaction to peptide formation in aqueous environments is the only reaction in which the free energy barrier of the rate-determining step is higher in NCW, thereby disfavoring decomposition of the formed peptide under nanoconfinement conditions compared to both ambient and hot-pressurized bulk water. As explained in the discussion part of the main text, the underlying reason is nanoconfinement-induced steric hindrance because the hydrolyzing water molecule must attack an atom at the center of the dipeptide (Fig. 12), which is however shielded due to the confining surfaces in conjunction with closeby parts of the peptide chain (Fig. 5 of the main text).

[1] Wittekindt, C. & Marx, D. Water confined between sheets of mackinawite FeS minerals. *J. Chem. Phys.* **137**, 054710 (2012).

[2] Muñoz-Santiburcio, D., Wittekindt, C. & Marx, D. Nanoconfinement effects on hydrated excess protons in layered materials. *Nat. Commun.* **4**, 2349 (2013).

[3] Muñoz-Santiburcio, D. & Marx, D. On the complex structural diffusion of proton holes in nanoconfined alkaline solutions within slit pores. *Nat. Commun.* **7**, 12625 (2016).

[4] Nair, N. N., Schreiner, E. & Marx, D. Peptide synthesis in aqueous environments: The role of extreme conditions on amino acid activation. *J. Am. Chem. Soc.* **130**, 14148–14160 (2008).

[5] Schreiner, E., Nair, N. N. & Marx, D. Peptide synthesis in aqueous environments: The role of extreme conditions on peptide bond formation and peptide hydrolysis. *J. Am. Chem. Soc.* **131**, 13668–13675 (2009).

[6] Bonthuis, D. J., Gekle, S. & Netz, R. R. Dielectric profile of interfacial water and its effect on double-layer capacitance. *Phys. Rev. Lett.* **107**, 166102 (2011).

[7] Bonthuis, D. J., Gekle, S. & Netz, R. R. Profile of the static permittivity tensor of water at interfaces: Consequences for capacitance, hydration interaction and ion adsorption. *Langmuir* **28**, 7679–7694 (2012).

[8] Marx, D. & Hutter, J. *Ab Initio Molecular Dynamics: Basic Theory and Advanced Methods* (Cambridge University Press, Cambridge, 2009).

[9] Perdew, J. P., Burke, K. & Ernzerhof, M. Generalized gradient approximation made simple. *Phys. Rev. Lett.* **77**, 3865–3868 (1996). Erratum: Phys. Rev. Lett. **78**, 1396–1396 (1997).

[10] Vanderbilt, D. Soft self-consistent pseudopotentials in a generalized eigenvalue formalism. *Phys. Rev. B* **41**, 7892–7895 (1990).

[11] Pollet, R., Boehme, C. & Marx, D. Ab initio simulations of desorption and reactivity of glycine at a water-pyrite interface at "Iron-Sulfur world" prebiotic conditions. *Origins Life Evol. B.* **36**, 363–379 (2006).

[12] Nair, N. N., Schreiner, E. & Marx, D. Glycine at the pyrite-water interface: The role of surface defects. *J. Am. Chem. Soc.* **128**, 13815–13826 (2006).

[13] Grimme, S., Antony, J., Ehrlich, S. & Krieg, H. A consistent and accurate ab initio parametrization of density functional dispersion correction (DFT-D) for the 94 elements H-Pu.

J. Chem. Phys. **132**, 154104 (2010).

[14] Sharma, M., Resta, R. & Car, R. Dipolar correlations and the dielectric permittivity of water. *Phys. Rev. Lett.* **98**, 247401 (2007).

[15] Pan, D., Spanu, L., Harrison, B., Sverjensky, D. A. & Galli, G. Dielectric properties of water under extreme conditions and transport of carbonates in the deep earth. *Proc. Natl. Acad. Sci. U.S.A.* **110**, 6646–6650 (2013).

[16] Grimme, S. Improved second-order Møller-Plesset perturbation theory by separate scaling of parallel- and antiparallel-spin pair correlation energies. *J. Chem. Phys.* **118**, 9095–9102 (2003).

[17] Weigend, F., Häser, M., Patzelt, H. & Ahlrichs, R. RI-MP2: optimized auxiliary basis sets and demonstration of efficiency. *Chem. Phys. Lett.* **294**, 143–152 (1998).

[18] Takatani, T. & Sherrill, C. D. Performance of spin-component-scaled Møller–Plesset theory (SCS-MP2) for potential energy curves of noncovalent interactions. *Phys. Chem. Chem. Phys.* **9**, 6106–6114 (2007).

[19] Weigend, F. & Ahlrichs, R. Balanced basis sets of split valence, triple zeta valence and quadruple zeta valence quality for H to Rn: Design and assessment of accuracy. *Phys. Chem. Chem. Phys.* **7**, 3297–3305 (2005).

[20] TURBOMOLE V6.2 2010, a development of University of Karlsruhe and Forschungszentrum Karlsruhe GmbH, 1989-2007, TURBOMOLE GmbH, since 2007; available from `http://www.turbomole.com`.

[21] Car, R. & Parrinello, M. Unified approach for molecular dynamics and density-functional theory. *Phys. Rev. Lett.* **55**, 2471 (1985).

[22] Martyna, G. J., Klein, M. L. & Tuckerman, M. Nosé-Hoover chains: The canonical ensemble via continuous dynamics. *J. Chem. Phys.* **97**, 2635–2643 (1992).

[23] Hutter, J. *et al.* *CPMD Program Package version 3.17.1.* IBM Corp 1990-2004, MPI für Festkörperforschung Stuttgart 1997-2001. See also `http://www.cpmd.org`.

[24] Marx, D., Tuckerman, M. E. & Parrinello, M. Solvated excess protons in water: quantum effects on the hydration structure. *J. Phys.: Condens. Matter* **12**, A153 (2000).

[25] Laio, A. & Parrinello, M. Escaping free-energy minima. *Proc. Natl. Acad. Sci.* **99**, 12562–12566 (2002).

[26] Laio, A. & Parrinello, M. Computing free energies and accelerating rare events with metady-

namics. In *Computer Simulations in Condensed Matter Systems: From Materials to Chemical Biology Volume 1*, 315–347 (Springer, 2006).

[27] Iannuzzi, M., Laio, A. & Parrinello, M. Efficient exploration of reactive potential energy surfaces using Car-Parrinello molecular dynamics. *Phys. Rev. Lett.* **90**, 238302 (2003).

[28] Bussi, G., Laio, A. & Parrinello, M. Equilibrium Free Energies from Nonequilibrium Metadynamics. *Phys. Rev. Lett.* **96**, 10–13 (2006).

[29] Raiteri, P., Laio, A., Gervasio, F. L., Micheletti, C. & Parrinello, M. Efficient reconstruction of complex free energy landscapes by multiple walkers metadynamics. *J. Phys. Chem. B* **110**, 3533–3539 (2006).

[30] Nair, N. N., Schreiner, E. & Marx, D. *inSiDE* **6**, 30–35 (2008). See: http://inside.hlrs.de/htm/Edition_02_08/article_09.html (accessed on February 25, 2015).

[31] Jülich Supercomputing Centre. JUQUEEN: IBM Blue Gene/Q® Supercomputer System at the Jülich Supercomputing Centre. *Journal of large-scale research facilities* **1**, A1 (2015).

[32] Sprik, M. Computation of the pK of liquid water using coordination constraints. *Chem. Phys.* **258**, 139–150 (2000).

[33] Ensing, B., Laio, A., Parrinello, M. & Klein, M. L. A recipe for the computation of the free energy barrier and the lowest free energy path of concerted reactions. *J. Phys. Chem. B* **109**, 6676–6687 (2005).

[34] Onufriev, A. Continuum electrostatics solvent modeling with the Generalized Born model. In *Modeling Solvent Environments*, 127–165 (Wiley-VCH Verlag GmbH & Co. KGaA, 2010).

[35] Truhlar, D. G. & Pliego, J. R. Transition state theory and chemical reaction dynamics in solution. In *Continuum Solvation Models in Chemical Physics*, 338–365 (John Wiley & Sons, Ltd, 2007).

[36] Neumann, M. & Steinhauser, O. Computer simulation and the dielectric constant of polarizable polar systems. *Chem. Phys. Lett.* **106**, 563–569 (1984).

[37] Stern, H. A. & Feller, S. E. Calculation of the dielectric permittivity profile for a nonuniform system: application to a lipid bilayer simulation. *J. Chem. Phys.* **118**, 3401–3412 (2003).

[38] Ballenegger, V. & Hansen, J.-P. Dielectric permittivity profiles of confined polar fluids. *J. Chem. Phys.* **122**, 114711 (2005).

[39] Zhang, C., Gygi, F. & Galli, G. Strongly anisotropic dielectric relaxation of water at the nanoscale. *J. Phys. Chem. Lett.* **4**, 2477–2481 (2013).

[40] Schlaich, A., Knapp, E. W. & Netz, R. R. Water dielectric effects in planar confinement. *Phys. Rev. Lett.* **117**, 048001 (2016).

[41] Schaaf, C. & Gekle, S. Dielectric response of the water hydration layer around spherical solutes. *Phys. Rev. E* **92**, 032718 (2015).

[42] Hünenberger, P. & Reif, M. *Single-Ion Solvation: Experimental and Theoretical Approaches to Elusive Thermodynamic Quantities* (RSC Publishing, Cambridge, 2011).

[43] Fernández, D. P., Goodwin, A. R. H., Lemmon, E. W., Levelt Sengers, J. M. H. & Williams, R. C. A formulation for the static permittivity of water and steam at temperatures from 238 K to 873 K at pressures up to 1200 MPa, including derivatives and Debye–Hückel coefficients. *J. Phys. Chem. Ref. Data* **26**, 1125–1166 (1997).
